# New Urea Derivatives as Potential Antimicrobial Agents: Synthesis, Biological Evaluation, and Molecular Docking Studies

**DOI:** 10.3390/antibiotics8040178

**Published:** 2019-10-09

**Authors:** Mahadev Patil, Anurag Noonikara-Poyil, Shrinivas D. Joshi, Shivaputra A. Patil, Siddappa A. Patil, Alejandro Bugarin

**Affiliations:** 1 Centre for Nano and Material Sciences, Jain University, Jain Global Campus, Bangalore 562112, Karnataka, India; 2 Department of Chemistry and Biochemistry, The University of Texas at Arlington, Arlington, TX 76019, USA; 3 Department of Pharmaceutical Chemistry, S. E. T’s College of Pharmacy, Sangolli Rayanna Nagar, Dharwad 580 002, Karnataka, India; 4 Pharmaceutical Sciences Department, College of Pharmacy, Rosalind Franklin University of Medicine and Science, North Chicago, IL 60064, USA; 5 Department of Chemistry and Physics, Florida Gulf Coast University, Fort Myers, FL 33965, USA

**Keywords:** urea, synthesis, antimicrobial activity, molecular docking, *Acinetobacter baumannii*

## Abstract

A series of new urea derivatives, containing aryl moieties as potential antimicrobial agents, were designed, synthesized, and characterized by ^1^H NMR, ^13^C NMR, FT-IR, and LCMS spectral techniques. All newly synthesized compounds were screened in vitro against five bacterial strains (*Escherichia coli, Klebsiella pneumoniae, Acinetobacter baumannii, Pseudomonas aeruginosa,* and *Staphylococcus aureus*) and two fungal strains (*Candida albicans* and *Cryptococcus neoformans*). Variable levels of interaction were observed for these urea derivatives. However, and of major importance, many of these molecules exhibited promising growth inhibition against *Acinetobacter baumannii*. In particular, to our delight, the adamantyl urea adduct **3l** demonstrated outstanding growth inhibition (94.5%) towards *Acinetobacter baumannii*. In light of this discovery, molecular docking studies were performed in order to elucidate the binding interaction mechanisms of the most active compounds, as reported herein.

## 1. Introduction

Bacterial and fungal diseases have become increasingly more prominent and complex in recent years, particularly when compared to the last half of the twentieth century [1]. As a result, the need for new antibacterial and antifungal agents has become a goal of extreme importance, especially in light of the documented emergence of multi-drug-resistant (MDR) strains in recent years [2,3,4,5,6,7]. These MDR strains already pose a well-recognized health threat to the world population and are frequently associated with increased healthcare cost and prolonged hospital stays. Despite recent advances to understanding the pathogenesis of infection, research laboratories have become increasingly focused on the discovery of new and more effective drug candidates as the MDR strains continue to increase. In particular, various, novel, synthetic small molecules have been synthesized and screened as potential candidates to combat these resistant strains of bacteria and fungi [8,9,10,11,12]. We have been actively involved in the design and discovery of such new bioactive molecules to tackle MDR strains [13,14,15,16,17,18,19,20,21,22] and have reported our most recent studies herein, which include some very promising discoveries.

**Figure 1:**
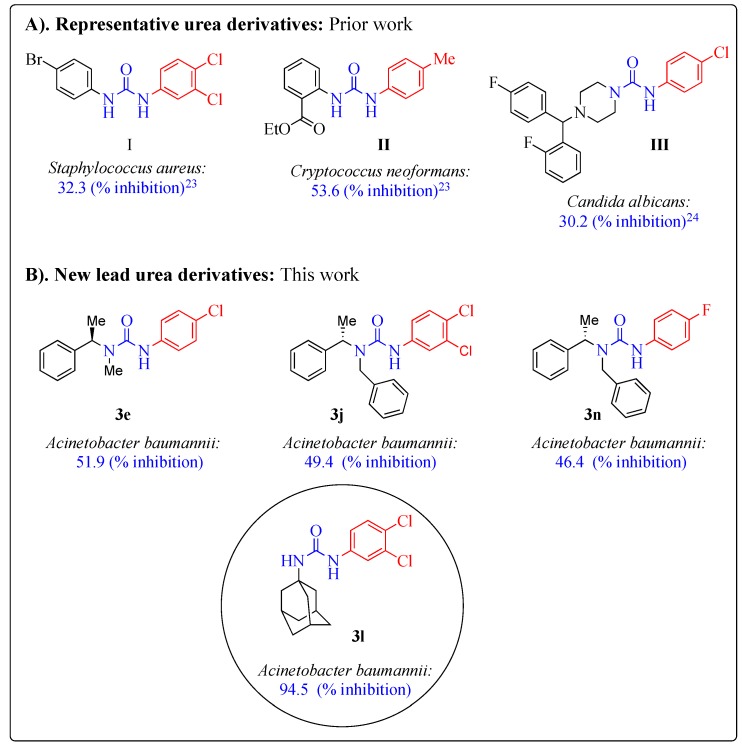
Antimicrobial urea derivatives.

Since urea functionality is a core moiety in many drug candidates, we have recently commenced a research program focused on the development of potential urea functionalized antimicrobial agents [23]. Our initial research efforts have identified the novel urea derivatives I and II as the initial hits for bacterial strain *Staphylococcus aureus* and fungal strain *Cryptococcus neoformans,* respectively, (Figure 1A) [23]. We have also studied the antimicrobial effects of *N,N*-disubstituted urea derivatives, where one of the urea nitrogen was part of a piperazine ring system (e.g., III which showed moderate inhibition against *Candida albicans* [24]. Encouraged by these results, we embarked on further exploring novel new urea derivatives for antimicrobial activities, as shown in Figure 1B.

**Scheme 1:**
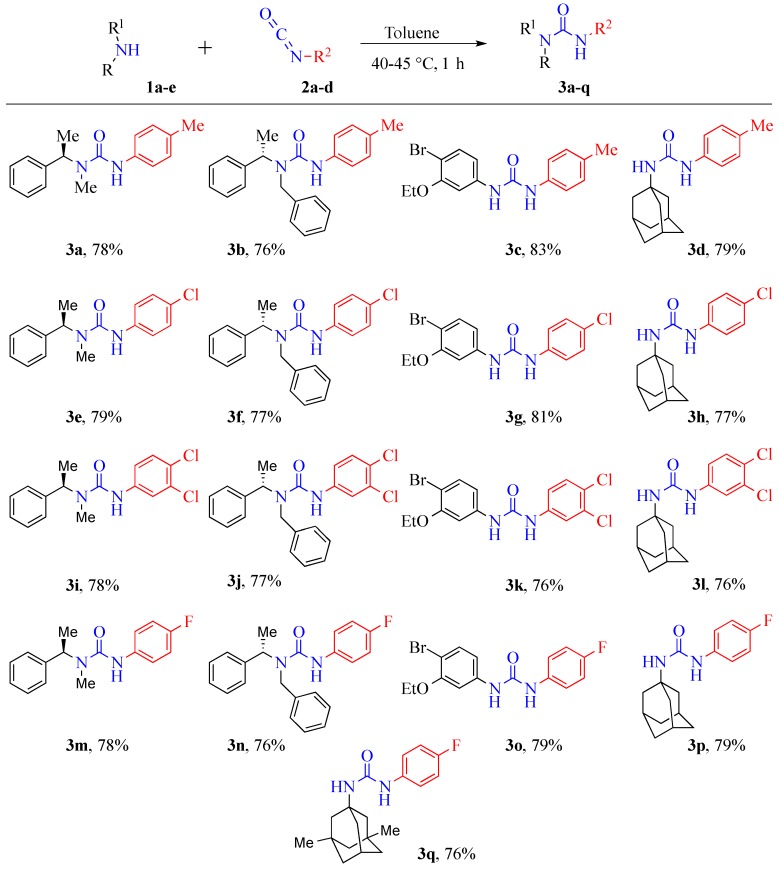
Synthesis of urea derivatives (**3a**–**q**).

In this work, we explored the effects of diverse substitutions present on the nitrogen atoms of the urea backbone. For instance, aryl or aliphatic substitutions were studied (Scheme 1). Very interestingly, *N,N*-disubstituted compounds; (*R*)-3-(4-chlorophenyl)-1-methyl-1-(1-phenylethyl)urea (3e), (*S*)-1-benzyl-3-(3,4-dichlorophenyl)-1-(1-phenylethyl)urea (3j), and (S)-1-benzyl-3-(4-fluorophenyl)-1-(1-phenylethyl)urea (3n) demonstrated a good inhibition towards *Acinetobacter baumannii*. Remarkably, this study identified a new potential lead drug candidate, 1-((3*S*,5*S*,7*S*)-adamantan-1-yl)-3-(3,4-dichlorophenyl)urea (3l) for *Acinetobacter baumannii*. This compound (3l) exhibited selective and outstanding inhibition (94.5%) towards *Acinetobacter baumannii*. A unique feature of this compound was that it included the lipophilic adamantane moiety (Figure 1). Notably, this adamantane functionality is extensively used in drug design and is part of several drug candidates [25,26,27,28]. 

## 2. Results and Discussion

### 2.1. Chemistry

The basic synthetic approach for preparation of the seventeen new urea derivatives, containing aryl moieties, is illustrated in Scheme 1. All compounds (**3a**–**q**) were successfully synthesized in a simple, one-step method, via the reaction of amines with commercially available isocyanates, at 40–45 °C, in toluene. The attractive features of this method were, (1) simple one-pot procedure, (2) mild reaction conditions, (3) short reaction time, (4) easier work-up, and (5) high yields (76% to 83%).

### 2.2. Spectroscopic Characterization

The chemical structures of the reported urea derivatives (**3a**–**q**) were confirmed by ^1^H NMR, ^13^C NMR, FT-IR, and LCMS spectral techniques. The spectral data of the compounds (**3a**–**q**) are presented in the experimental section; these were in accordance with assigned structures for urea derivatives containing aryl moieties (Scheme 1). The FT–IR spectra, for the title urea compounds, were recorded within the region of 400 to 4000 cm^−1^. The IR spectra of all urea derivatives exhibited bands at 3264–3374 and 1648–1626 cm^−1^ assigned to the ν(NH) and ν(C=O) groups, respectively. IR observed signals at 3028–2905 and 1595–1504 cm^−1^ might be assigned to the C-H and C=C stretches of the aromatic rings. The chemical structure of all molecules was further confirmed by ^1^H NMR spectroscopy and further confirmed by the ^13^C NMR spectra. The ^13^C NMR spectra of all urea derivatives (**3a**–**q**) exhibited a characteristic signal of the urea carbonyl functional group (C=O) between 157.5 and 153.5 ppm. Further confirmation of the molecular structures of the reported compounds was assessed by mass spectra. The molecular ion peak (M + H)^+^ and the base peak for all urea compounds were clearly found in the mass spectrometry study. Thus, the molecular ion peaks agreed with the molecular weight of the respective compounds.

### 2.3. Antimicrobial Activity

The in vitro antimicrobial activity of the freshly produced urea derivatives (**3a**–**q**) was evaluated against five bacteria [*Escherichia coli, Klebsiella pneumoniae, Acinetobacter baumannii, Pseudomonas aeruginosa,* and *Staphylococcus aureus*] and two fungal [*Candida albicans* and *Cryptococcus neoformans*] species. Colistin was used as a positive inhibitor standard for Gram-negative bacteria; Vancomycin was used for the Gram-positive bacteria; and Fluconazole was used as a positive fungal inhibitor standard for both fungi. The antimicrobial potential/results of these urea derivatives are summarized in Table 1.

The antimicrobial screening disclosed that some of the tested compounds demonstrated excellent to moderate growth inhibition towards various tested microbial strains. The result indicated that among the tested compounds, compounds **3c**, **3e**, **3f**, **3i**, **3j**, **3l**, and **3n** showed moderate to excellent growth inhibition against *A. baumannii*. Further, it was found that compounds (**3c** and **3g**) showed moderate growth inhibition towards *K. pneumonia,* whereas compound (**3k**) demonstrated moderate growth inhibition against *S. aureus*. In contrast, all urea derivatives screened (**3a**–**q**) against *E. coli*, *P. aeruginosa*, and *C. albicans* exhibited moderate to poor growth inhibition. Compound (**3a**) showed moderate growth inhibition against the *C. neoformans* fungi, whereas the remaining compounds (**3b**–**q**) revealed a poor growth inhibition. Overall, the results indicated that the adamantyl urea derivative (**3l**) showed the highest growth inhibition towards the *A. baumannii* bacteria (94.5%) (Table 1), a very exciting observation, which suggested development and biological evaluation of other adamantyl urea derivatives.

### 2.4. Molecular Docking Studies

**Figure 2:**
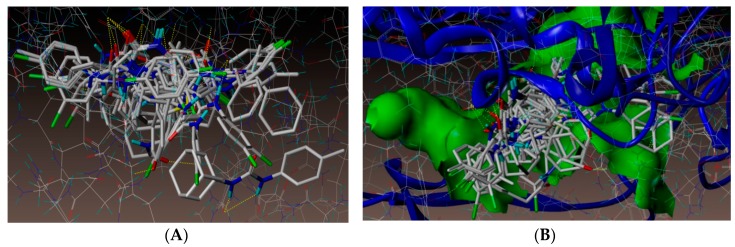
(**A**) All compounds docked into the active site of enoyl-(acyl-carrier-protein) reductase (ENR). (**B**) Docked view of all the compounds at the active site of enzyme PDB ID: 3UDI.

As noted above, antimicrobial screening revealed that our urea derivatives were superior inhibitors against *A. baumannii*. Therefore, to investigate the mechanism of this antibacterial action, and explore the intermolecular interactions between the synthesized compounds with the receptor, molecular docking studies were performed on the crystal structure of the *A. baumannii* PBP1a, in complex with penicillin G (PDB ID 3UDI, 2.6 Å X-ray resolution), using the surflex-dock program of the sybyl-X 2.0 software. All inhibitors, along with the ligand, were docked into the active site of enoyl-(acyl-carrier-protein) reductase (ENR), as shown in Figure 2. The predicted binding energies of the compounds are listed in Table 2. The docking study revealed that all the compounds exhibited very good docking scores against the enzyme.

**Figure 3:**
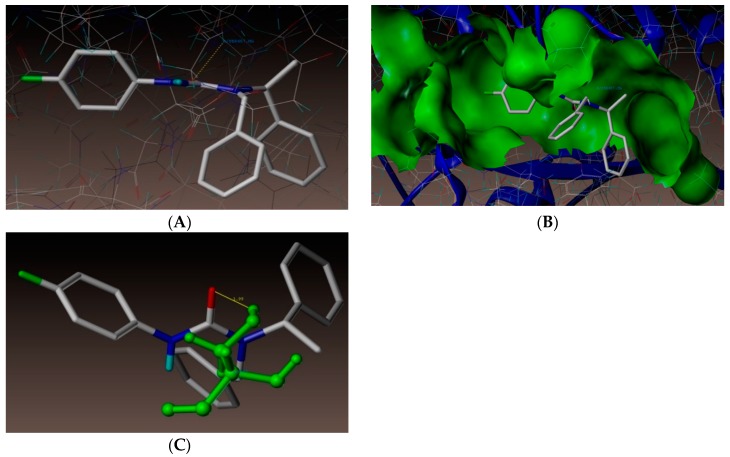
(**A**) Docked view of compound **3n**. (**B**) Docked view of compound **3n** at the active site of enzyme PDB ID: 3UDI. (**C**) Hydrogen-bond interaction of compound **3n** at the active site.

**Figure 4:**
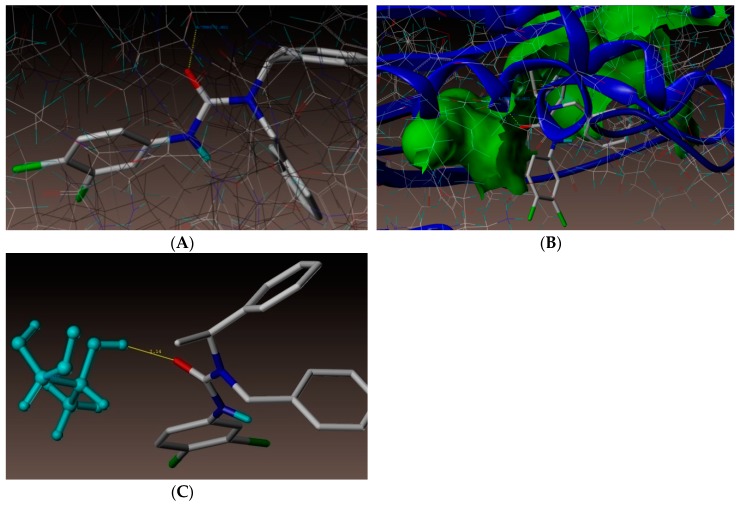
(**A**) Docked view of compound **3j**. (**B**) Docked view of compound **3j** at the active site of enzyme PDB ID: 3UDI. (**C**) Hydrogen-bond interaction of compound **3j** at the active site.

**Figure 5:**
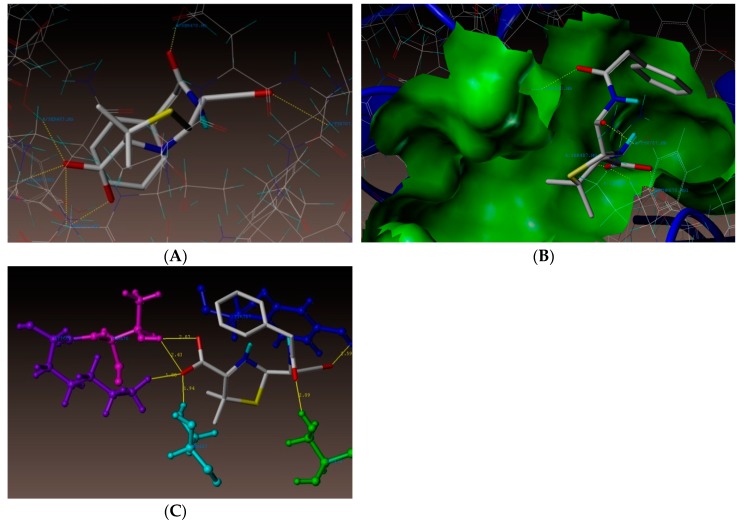
(**A**) Docked views of 3UDI_ligand. (**B**) Docked view of 3UDI_ligand at the active site of enzyme PDB ID: 3UDI. (**C**) Neighboring interactions of 3UDI_ligand.

**Figure 6:**
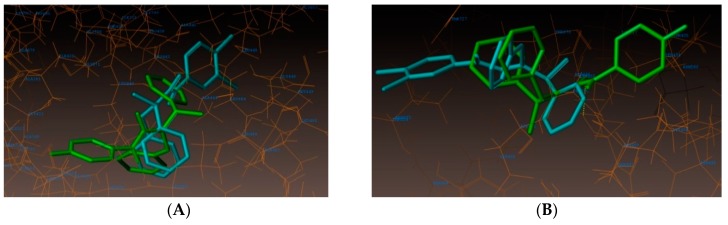
(**A**) Hydrophobic amino acids surrounded to compounds **3n** (green color) and **3j** (cyan color). (**B**) Hydrophilic amino acids surrounded by compounds **3n** and **3j**.

As depicted in Figure 3, compound (**3n**) makes a hydrogen bonding interaction at the active site of the enzyme (PDB ID: 3UDI), the oxygen atom of carbonyl group interacts with the hydrogen atom of SER487 (C=O------H-SER487, 1.99 Å). As depicted in Figure 4, compound (**3j**) makes a hydrogen bonding interaction at the active site of the enzyme (PDB ID: 3UDI), oxygen atom of the carbonyl group interacts with the hydrogen atom of THR670 (C=O------H-THR670, 2.14 Å). The binding interaction of 3UDI_ligand with enzyme active sites shows six bonding interactions and the docked view of the same; as depicted in Figure 5. Figure 6A,B represents the hydrophobic and hydrophilic amino acids surrounded to the studied compound (**3n** and **3j**).

All compounds showed consensus scores in the range 5.19–2.91, indicating the summary of all forces of interaction between ligands and the enzyme. Moreover, it was observed that the studied compounds displayed the same type of interactions with amino acid residues SER487 and THR670, as that of the reference 3UDI_ligand. This indicated that molecules preferentially bound to the enzyme in comparison to the reference 3UDI_ligand (Table 2).

**Figure 7:**
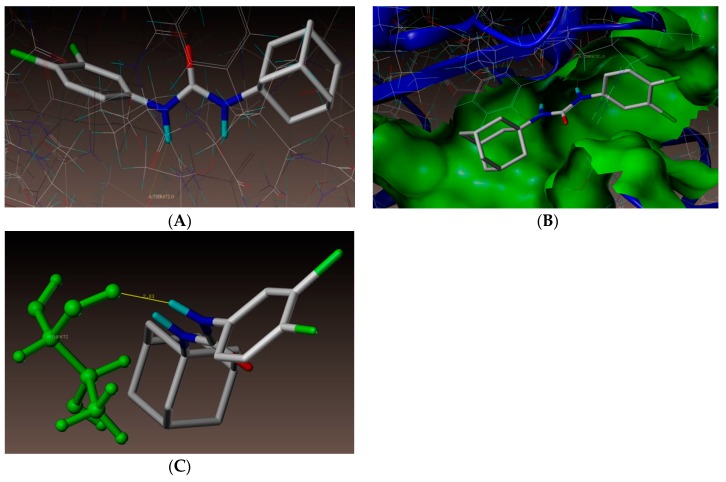
(**A**) Docked view of compound **3l**. (**B**) Docked view of compound **3l** at the active site of enzyme PDB ID: 3UDI. (**C**) Hydrogen-bond interaction of compound **3l** at the active site.

**Figure 8:**
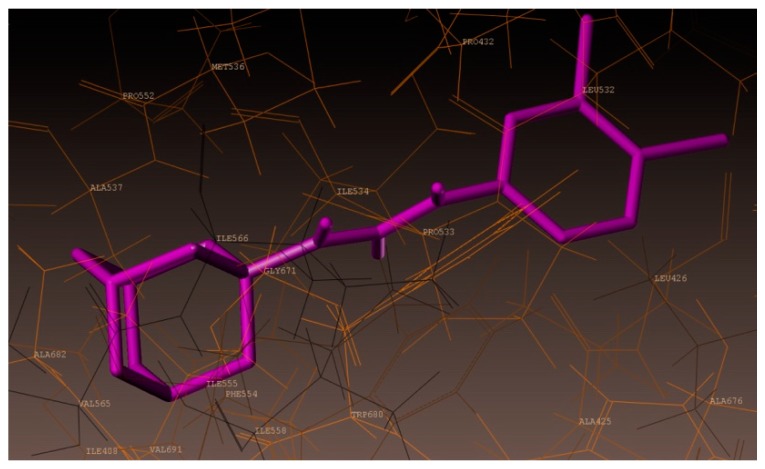
Hydrophobic amino acids surrounding compound **3l** (magenta color).

**Figure 9:**
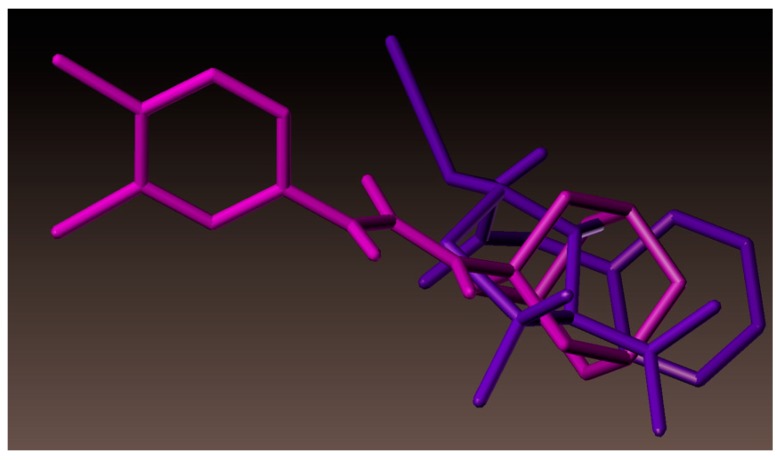
Superimposition of compound **3l** (magenta color) with benzyl penicillin (purple color).

As documented above, new urea derivative (**3l**) showed highest growth inhibition against *A. baumannii*. Hence, a molecular docking study was also performed for **3l**. As represented in Figure 7, compound **3l** showed hydrogen bonding interaction at the active site of the enzyme (PDB ID: 3UDI). The adamantane ring was surrounded by hydrophobic amino acids ALA537, ILE566, GLY675, ILE555, PHE554, ALA682, and VAL565 (Figure 8), which might have resulted in the increased activity of compound (**3l**). Superimposition of **3l** (magenta color) with benzyl penicillin (purple color) has been depicted in Figure 9. As shown in the figure, the adamantane moiety was superimposed with β-lactam and thiazolidine rings. 

## 3. Materials and Methods 

### 3.1. General Consideration

All chemicals, including isocyanates, were purchased from Sigma-Aldrich chemical company and were used without further purification. All solvents were of analytical grade and were used without further purification. All reactions were carried out under aerobic conditions, in oven-dried glassware with magnetic stirring. Heating was accomplished by either a heating mantle or silicone oil bath. Reactions were monitored by thin-layer chromatography (TLC) performed on 0.25 mm Merck TLC silica gel plates, using UV light as a visualizing agent. Purification of the reaction products was carried out by flash column chromatography using silica gel 60 (230–400 mesh). Yields refer to the chromatographically pure material. Concentration in vacuo refers to the removal of volatile solvent using a rotary evaporator attached to a dry diaphragm pump (10–15 mm Hg), followed by pumping to a constant weight with an oil pump (<300 mTorr). ^1^H spectra were recorded on JEOL Eclipse Plus 500 (500 MHz) and were reported relative to CDCl_3_ (δ 7.26) or DMSO-*d*_6_ (δ 2.50). ^1^H NMR coupling constants (J) were reported in Hertz (Hz) and multiplicities were indicated as follows—s (singlet), d (doublet), t (triplet), quint (quintet), m (multiplet). Proton-decoupled ^13^C NMR spectra were recorded on the JEOL Eclipse Plus 500 (125 MHz) and were reported relative to CDCl_3_ (δ 77.00) or DMSO-*d*_6_ (δ 39.52). IR spectra were recorded on an Alpha-P Bruker FT/IR spectrometer. Liquid chromatography mass spectra (LC-MS) were recorded on Agilent technologies quadrupole LC–MS system.

### 3.2. Syntheses

#### 3.2.1. General Experimental Procedure for the Synthesis of Urea Derivatives

To a solution of isocyanate (1.877 mmol) in toluene (2.5 mL) a solution of amine (1.877 mmol) in toluene (1.0 mL) was added. The reaction mixture was heated at 40–45 °C for 1 h. Then, the reaction mixture was cooled down to the room temperature. The resulting solids were filtered and washed with toluene (2.0 mL). Additional toluene (2.5 mL) was added to the solids and stirred at room temperature for about 30 minutes, filtered, and washed with more toluene (2.0 mL) to obtain the crude urea derivatives. Finally, the crude urea derivatives were purified by silica gel flash column chromatography, using hexane/ethyl acetate (9:1) as the eluents, to afford pure adducts. ^1^H and ^13^C NMR data of all the compounds (**3a**–**q**) in Supplementary Materials.

##### Synthesis of (R)-1-methyl-1-(1-phenylethyl)-3-(p-tolyl)urea (**3a**)

Compound (**3a**) was synthesized from 4-methylphenyl isocyanate (0.25 g, 1.87 mmol) and (R)-(+)-N, α-dimethylbenzylamine (0.25 g, 1.87 mmol), according to the general procedure. It was a white solid; yield— 78% (0.39 g). ^1^H NMR (CDCl_3_, 500 MHz): δ 7.39–7.36 (m, 4H), 7.29 (d, *J* = 8.0 Hz, 3H), 7.09 (d, *J* = 8.0 Hz, 2H), 6.53 (br, 1H), 5.70 (q, *J* = 6.9 Hz, 1H), 2.73 (s, 3H), 2.30 (s, 3H), 1.56 (d, *J* = 6.9 Hz, 3H). ^13^C NMR (CDCl_3_, 125 MHz): δ 155.5, 140.9, 136.3, 132.6, 129.3, 128.5, 127.3, 127.1, 120.0, 52.6, 29.4, 20.7, 16.6. IR (KBr): ῡ = 3304.2, 2923.4, 1633.9, 1595.6, 1291.7, 811.2.LC-MS for C_17_H_20_N_2_O (268.35): *m*/*z* = 269.32 [M + H]^+^.

##### Synthesis of (S)-1-benzyl-1-(1-phenylethyl)-3-(p-tolyl)urea (**3b**)

Compound (**3b**) was synthesized from 4-methylphenyl isocyanate (0.25 g, 1.87 mmol) and (S)-(-)-N-benzyl-α-methylbenzylamine (0.39 g, 1.87 mmol), according to the general procedure. It was a white solid; yield—76% (0.49 g). ^1^H NMR (DMSO-*d*_6_, 500 MHz): δ 8.26 (s, 1H), 7.38–7.33 (m, 4H), 7.30 (d, *J* = 8.6 Hz, 2H), 7.28–7.24 (m, 3H), 7.20–7.15 (m, 3H),7.02 (d, *J* = 8.0 Hz, 2H), 5.72 (q, *J* = 7.5 Hz, 1H), 4.68 (d, *J* = 17.8 Hz, 1H), 4.16 (d, *J* = 17.2 Hz, 1H), 2.21 (s, 3H), 1.44 (d, *J* = 7.5 Hz, 3H). ^13^C NMR (DMSO-*d*_6_, 125 MHz): δ 155.7, 142.0, 139.9, 137.8, 130.7, 128.7, 128.4, 128.1, 127.1, 127.0, 126.5, 126.4, 120.0, 52.9, 45.4, 20.3, 17.9. IR (KBr): ῡ = 3353.6, 2918.3, 1633.2, 1511.5, 1239.1, 816.3. LC-MS for C_23_H_24_N_2_O (344.45): *m*/*z* = 345.43 [M + H]^+^.

##### Synthesis of 1-(4-bromo-3-ethoxyphenyl)-3-(p-tolyl)urea (**3c**)

Compound (**3c**) was synthesized from 4-methylphenyl isocyanate (0.25 g, 1.87 mmol) and 4-bromo-3-ethoxyaniline (0.40 g, 1.87 mmol), according to the general procedure. It was a white solid; yield—83% (0.54 g). ^1^H NMR (DMSO-*d*_6_, 500 MHz): δ 8.76 (s, 1H), 8.58 (s, 1H), 7.41 (d, *J* = 8.6 Hz, 1H), 7.39 (d, *J* = 2.9 Hz, 1H), 7.33 (d, *J* = 8.6 Hz, 2H), 7.08 (d, *J* = 8.0 Hz, 2H), 6.87 (dd, *J* = 8.6, 2.3 Hz, 1H), 4.06 (q, *J* = 6.9 Hz, 1H), 2.24 (s, 3H), 1.36 (t, *J* = 7.6 Hz, 1H). ^13^C NMR (DMSO-*d*_6_, 125 MHz): δ 154.8, 152.5, 140.7, 136.9, 132.6, 130.9, 129.2, 118.5, 111.4, 103.6, 102.4, 64.1, 20.4, 14.6. IR (KBr): ῡ = 3340.0, 2983.6, 1648.7, 1321.5, 1045.4, 821.9. LC-MS for C_16_H_17_BrN_2_O_2_ (349.22): *m*/*z* = 350.21 [M + H]^+^.

##### Synthesis of 1-((3s,5s,7s)-adamantan-1-yl)-3-(p-tolyl)urea (**3d**)

Compound (**3d**) was synthesized from 4-methylphenyl isocyanate (0.25 g, 1.87 mmol) and 1-amino adamantane (0.28 g, 1.87 mmol), according to the general procedure. It was a white solid; yield—79% (0.42 g). ^1^H NMR (DMSO-*d*_6_, 500 MHz): δ 8.11 (s, 1H), 7.21 (d, *J* = 8.6 Hz, 2H), 6.99 (d, *J* = 8.0 Hz, 2H), 5.80 (s, 1H), 2.20 (s, 3H), 2.02 (s, 3H), 1.95–1.90 (m, 6H), 1.62 (s, 6H). ^13^C NMR (DMSO-*d*_6_, 125 MHz): δ 154.1, 138.1, 129.4, 129.0, 117.4, 49.8, 41.7, 36.1, 28.9, 20.3. IR (KBr): ῡ = 3324.0, 2908.2, 2884.3, 1642.4, 1556.6, 1235.8, 810.3.LC-MS for C_18_H_24_N_2_O (284.40): *m*/*z* = 285.39 [M + H]^+^.

##### Synthesis of (R)-3-(4-chlorophenyl)-1-methyl-1-(1-phenylethyl)urea (**3e**)

Compound (**3e**) was synthesized from 4-chlorophenyl isocyanate (0.25 g, 1.62 mmol) and (R)-(+)-N, α-dimethylbenzylamine (0.22 g, 1.62 mmol), according to the general procedure. It was a white solid; yield—79% (0.37 g). ^1^H NMR (DMSO-*d*_6_, 500 MHz): δ 8.48 (s, 3H), 7.59–7.56 (m, 2H), 7.36 (t, *J* = 7.5 Hz, 2H), 7.33–7.25 (m, 5H), 5.64 (q, *J* = 6.9 Hz, 1H), 2.67 (s, 3H), 1.48 (d, *J* = 6.9 Hz, 3H). ^13^C NMR (DMSO-*d*_6_, 125 MHz): δ 155.5, 141.5, 139.7, 128.4, 128.1, 126.9, 126.8, 125.3, 121.3, 51.3, 28.7, 16.4. IR (KBr): ῡ = 3306.3, 3028.5, 1638.3, 1518.7, 1241.5, 821.0. LC-MS for C_16_H_17_ClN_2_O (288.77): *m*/*z* = 289.65 [M + H]^+^.

##### Synthesis of (S)-1-benzyl-3-(4-chlorophenyl)-1-(1-phenylethyl)urea (**3f**)

Compound (**3f**) was synthesized from 4-chlorophenyl isocyanate (0.25 g, 1.62 mmol) and (S)-(-)-N-benzyl-α-methylbenzylamine (0.34 g, 1.62 mmol), according to the general procedure. It was a white solid; yield—77% (0.45 g). ^1^H NMR (DMSO-*d*_6_, 500 MHz): δ 8.55 (s, 1H), 7.48 (d, *J* = 9.2 Hz, 2H), 7.38–7.33 (m, 4H), 7.28–7.33 (m, 5H), 7.18–7.15 (m, 3H), 5.71 (q, *J* = 7.5 Hz, 1H), 4.68 (d, *J* = 17.2 Hz, 1H), 4.17 (d, *J* = 17.2 Hz, 1H), 1.45 (d, *J* = 6.9 Hz, 3H). ^13^C NMR (DMSO-*d*_6_, 125 MHz): δ 155.5, 141.8, 139.6, 139.4, 128.4, 128.1 (2C), 127.1, 127.0, 126.4 (2C), 125.5, 121.3, 53.1, 45.5, 17.9. IR (KBr): ῡ =3373.7, 2972.8, 2934.0, 1636.8, 1520.7, 1232.3, 829.9. LC-MS for C_22_H_21_ClN_2_O (364.87): *m*/*z* = 365.83 [M + H]^+^.

##### Synthesis of 1-(4-bromo-3-ethoxyphenyl)-3-(4-chlorophenyl)urea (**3g**)

Compound (**3g**) was synthesized from 4-chlorophenyl isocyanate (0.25 g, 1.62 mmol) and 4-bromo-3-ethoxyaniline (0.351g, 1.62 mmol), according to the general procedure. It was a white solid; yield—81% (0.48 g). ^1^H NMR (DMSO-*d*_6_, 500 MHz): δ 8.85 (s, 1H), 8.84 (s, 1H), 7.49 (d, *J* = 9.2 Hz, 2H), 7.43-7.38 (m, 2H), 7.32 (d, *J* = 9.2 Hz, 2H), 6.89 (dd, *J* = 8.6, 2.3 Hz, 1H), 4.06 (q, *J* = 6.9 Hz, 2H), 1.36 (q, *J* = 6.9 Hz, 3H). ^13^C NMR (DMSO-*d*_6_, 125 MHz): δ 154.8, 152.3, 140.4, 138.5, 132.6, 128.6, 125.6, 119.9, 111.6, 103.8, 102.8, 64.2, 14.6. IR (KBr): ῡ = 3295.0, 2984.5, 2887.0, 1645.0, 1555.8, 1196.8, 824.2. LC-MS for C_15_H_14_BrClN_2_O_2_ (369.64): *m*/*z* = 370.62 [M + H]^+^.

##### Synthesis of 1-((3S,5S,7S)-adamantan-1-yl)-3-(4-chlorophenyl)urea (**3h**)

Compound (**3h**) was synthesized from 4-chlorophenyl isocyanate (0.25 g, 1.62 mmol) and 1-amino adamantane (0.24 g, 1.62 mmol), according to the general procedure. It was a white solid; yield—77% (0.38 g). ^1^H NMR (DMSO-*d*_6_, 500 MHz): δ 8.37 (s, 1H), 7.36 (d, *J* = 8.6 Hz, 2H), 7.22 (d, *J* = 8.6 Hz, 2H), 5.88 (s, 1H), 2.01 (s, 3H), 1.95–1.90 (m, 6H), 1.62 (s, 6H). ^13^C NMR (DMSO-*d*_6_, 125 MHz): δ 153.8, 139.6, 128.4, 124.1, 118.8, 49.9, 41.6, 36.0, 28.9. IR (KBr): ῡ = 3327.3, 2905.4, 2847.8, 1644.5, 1554.0, 1231.6, 815.2.LC-MS for C_17_H_21_ClN_2_O (304.81): *m*/*z* = 305.78 [M + H]^+^.

##### Synthesis of (R)-3-(3,4-dichlorophenyl)-1-methyl-1-(1-phenylethyl)urea (**3i**)

Compound (**3i**) was synthesized from 3,4-dichlorophenyl isocyanate (0.25 g, 1.32 mmol) and (R)-(+)-N, α-dimethylbenzylamine (0.17 g, 1.32 mmol), according to the general procedure. It was a white solid; yield—78% (0.335 g). ^1^H NMR (DMSO-*d*_6_, 500 MHz): δ 8.64 (s, 1H), 7.93 (d, *J* = 2.3 Hz, 1H), 7.54 (dd, *J* = 8.6, 2.3 Hz, 1H), 7.47 (d, *J* = 9.2 Hz, 1H), 7.36 (t, *J* = 7.5 Hz, 2H), 7.30 (d, *J* = 7.5 Hz, 2H), 7.26 (d, *J* = 6.9 Hz, 1H), 5.63 (q, *J* = 6.9 Hz, 1H), 2.67 (s, 3H), 1.48 (d, *J* = 6.9 Hz, 3H). ^13^C NMR (DMSO-*d*_6_, 125 MHz): δ 155.2, 141.3, 141.0, 130.5, 130.1, 128.4, 127.0, 126.9, 122.9, 120.7, 119.5, 51.4, 28.8, 16.3. IR (KBr): ῡ = 3263.3, 2982.5, 1636.0, 1580.8, 1236.1, 817.8. LC-MS for C_16_H_16_Cl_2_N_2_O (323.22): *m*/*z* = 324.15 [M + H]^+^.

##### Synthesis of (S)-1-benzyl-3-(3,4-dichlorophenyl)-1-(1-phenylethyl)urea (**3j**)

Compound (**3j**) was synthesized from 3,4-dichlorophenyl isocyanate (0.25g, 1.32 mmol) and (S)-(-)-N-benzyl-α-methylbenzylamine (0.28g, 1.32 mmol), according to the general procedure. It was a pale yellow liquid; yYield—77% (0.40 g). ^1^H NMR (DMSO-*d*_6_, 500 MHz): δ 8.75 (s, 1H), 8.32 (s, 1H), 7.86 (s, 1H), 7.45 (d, *J* = 1.2 Hz, 2H), 7.38–7.32 (m, 4H), 7.29–7.24 (m, 3H), 7.16 (d, *J* = 8.6 Hz, 3H), 5.69 (q, *J* = 6.9 Hz, 1H), 4.68 (d, *J* = 17.2 Hz, 1H), 4.19 (d, *J* = 17.2 Hz, 1H), 1.45 (d, *J* = 7.5 Hz, 3H). ^13^C NMR (DMSO-*d*_6_, 125 MHz): δ 155.2, 141.6, 140.7, 139.4, 130.6, 130.1, 128.5, 128.2, 127.2, 127.0, 126.5, 126.4, 123.1, 120.7, 119.6, 53.2, 45.6, 17.8. IR (KBr): ῡ = 3330.0, 2976.4, 2876.1, 1638.8, 1510.2, 1232.4, 750.7. LC-MS for C_22_H_20_Cl_2_N_2_O (399.31): *m*/*z* = 400.27 [M + H]^+^.

##### Synthesis of 1-(4-bromo-3-ethoxyphenyl)-3-(3,4-dichlorophenyl)urea (**3k**)

Compound (**3k**) was synthesized from 3,4-dichlorophenyl isocyanate (0.25 g, 1.32 mmol) and 4-bromo-3-ethoxyaniline (0.28 g, 1.32 mmol), according to the general procedure. It was a white solid; yield—76% (0.405 g). ^1^H NMR (DMSO-*d*_6_, 500 MHz): δ 9.01 (s, 1H), 8.94 (s, 1H), 7.88 (d, *J* = 2.3 Hz, 1H), 7.50 (d, *J* = 9.2 Hz, 1H), 7.42 (d, *J* = 8.6 Hz, 1H), 7.38 (d, *J* = 2.3 Hz, 1H), 7.32 (dd, *J* = 8.9, 2.3 Hz, 1H), 6.89 (dd, J 8.6, 2.3 Hz, 1H), 4.06 (q, *J* = 6.9 Hz, 2H), 1.36 (t, *J* = 7.5 Hz, 3H). ^13^C NMR (DMSO-*d*_6_, 125 MHz): δ 154.8, 152.2, 140.2, 139.8, 132.7, 131.1, 130.6, 123.3, 119.4, 118.5, 111.7, 103.9, 103.0, 64.2, 14.6. IR (KBr): ῡ = 3292.3, 2982.6, 2928.9, 1638.4, 1544.2, 1231.2, 800.2. LC-MS for C_15_H_13_BrCl_2_N_2_O_2_ (404.09): *m*/*z* = 405.10 [M + H]^+^.

##### Synthesis of 1-((3S,5S,7S)-adamantan-1-yl)-3-(3,4-dichlorophenyl)urea (**3l**)

Compound (**3l**) was synthesized from 3,4-dichlorophenyl isocyanate (0.25 g, 1.32 mmol) and 1-amino adamantane (0.20 g, 1.32 mmol), according to the general procedure. It was a white solid; yield—76% (0.34 g). ^1^H NMR (DMSO-*d*_6_, 500 MHz): δ 8.55 (s, 1H), 7.83 (d, *J* = 2.3 Hz, 1H), 7.40 (d, *J* = 8.6 Hz, 1H), 7.11 (dd, *J* = 8.9, 1.7 Hz, 1H), 5.96 (s, 1H), 2.01 (s, 3H), 1.95–1.88 (m, 6H), 1.62 (s, 6H). ^13^C NMR (DMSO-*d*_6_, 125 MHz): δ 153.5, 140.8, 131.0, 130.4, 121.9, 118.3, 117.4, 50.1, 41.5, 36.0, 28.9. IR (KBr): ῡ = 3331.1, 2905.5, 2850.5, 1646.7, 1548.3, 1226.7, 808.5. LC-MS for C_17_H_20_Cl_2_N_2_O (339.26): *m*/*z* = 340.15 [M + H]^+^.

##### Synthesis of (R)-3-(4-fluorophenyl)-1-methyl-1-(1-phenylethyl)urea (**3m**)

Compound (**3m**) was synthesized from 4-fluorophenyl isocyanate (0.25 g, 1.82 mmol) and (R)-(+)-N, α-dimethylbenzylamine (0.24 g, 1.82 mmol), according to the general procedure. It was a white solid; yield—78% (0.38 g). ^1^H NMR (DMSO-*d*_6_, 500 MHz): δ 8.39 (s, 1H), 7.56–7.53 (m, 2H), 7.39–7.25 (m, 5H), 7.08 (t, *J* = 9.2 Hz, 2H), 5.66 (q, *J* = 6.9 Hz, 1H), 2.67 (s, 3H), 1.48 (d, *J* = 6.9 Hz, 3H). ^13^C NMR (DMSO-*d*_6_, 125 MHz): δ 157.4 (d, ^1^J_C,F_ = 238.7 Hz), 155.7, 141.6, 137.0, 128.4, 126.9, 126.8, 121.7 (d, ^3^J_C,F_ = 8.4 Hz), 114.7 (d, ^2^J_C,F_ = 22.8 Hz), 51.3, 28.7, 16.4. IR (KBr): ῡ = 3264.8, 3067.4, 1634.8, 1507.9, 1212.5, 827.7. LC-MS for C_16_H_17_FN_2_O (272.32): *m*/*z* = 273.30 [M + H]^+^.

##### Synthesis of (S)-1-benzyl-3-(4-fluorophenyl)-1-(1-phenylethyl)urea (**3n**)

Compound (**3n**) was synthesized from 4-fluorophenyl isocyanate (0.25 g, 1.82 mmol) and (S)-(-)-N-benzyl-α-methylbenzylamine (0.38 g, 1.82 mmol), according to the general procedure. It was a white solid; yield—76% (0.479 g). ^1^ H NMR (DMSO-*d*_6_, 500 MHz): δ 8.46 (s, 1H), 7.45 (dd, *J* = 8.6, 5.2 Hz, 2H), 7.36 (d, *J* = 4.0 Hz, 4H), 7.26 (t, *J* = 7.5 Hz, 3H), 7.22–7.16 (m, 3H), 7.06 (t, *J* = 9.2 Hz, 2H), 5.73 (q, *J* = 6.9 Hz, 1H), 4.70 (d, *J* = 17.2 Hz, 1H), 4.17 (d, *J* = 17.2 Hz, 1H), 1.45 (d, *J* = 6.9 Hz, 3H). ^13^C NMR (DMSO-*d*_6_, 125 MHz): δ 157.5 (d, ^1^J_C,F_ = 237.5 Hz), 155.7, 141.9, 139.8, 136.7, 128.4, 128.1, 127.1, 127.0, 126.4, 126.3, 121.7 (d, ^3^J_C,F_ = 7.2 Hz), 114.7 (d, ^2^J_C,F_ = 22.8 Hz), 53.0, 45.5, 17.9. IR (KBr): ῡ = 3306.6, 2929.4, 2875.6, 1633.9, 1507.4, 1208.3, 828.1. LC-MS for C_22_H_21_FN_2_O (348.41): *m*/*z* = 349.36 [M + H]^+^.

##### Synthesis of 1-(4-bromo-3-ethoxyphenyl)-3-(4-fluorophenyl)urea (**3o**)

Compound (**3o**) was synthesized from 4-fluorophenyl isocyanate (0.25 g, 1.82 mmol) and 4-bromo-3-ethoxyaniline (0.39 g, 1.82 mmol), according to the general procedure. It was a white solid; yield—79% (0.64 g). ^1^H NMR (DMSO-*d*_6_, 500 MHz): δ 8.81 (s, 1H), 8.73 (s, 1H), 7.48–7.45 (m, 2H), 7.41 (d, *J* = 8.6 Hz, 1H), 7.38 (d, *J* = 2.3 Hz, 1H), 7.14–7.09 (m, 2H), 6.89 (dd, *J* = 8.6, 2.3 Hz, 1H), 4.06 (q, *J* = 6.9 Hz, 2H), 1.36 (d, *J* = 6.9 Hz, 3H). ^13^C NMR (DMSO-*d*_6_, 125 MHz): δ 157.5 (d, ^1^J_C,F_ = 238.7 Hz), 154.8, 152.5, 140.6, 135.8, 132.6, 120.2 (d, ^3^J_C,F_ = 7.2 Hz), 115.3 (d, ^2^J_C,F_ = 22.8 Hz), 111.5, 103.7, 102.6, 64.2, 14.6. IR (KBr): ῡ = 3284.9, 2983.7, 2900.8, 1626.6, 1504.4, 1212.5, 805.5. LC-MS for C_15_H_14_BrFN_2_O_2_ (353.19): *m*/*z* = 354.16 [M + H]^+^.

##### Synthesis of 1-((3S,5S,7S)-adamantan-1-yl)-3-(4-fluorophenyl)urea (**3p**)

Compound (**3p**) was synthesized from 4-fluorophenyl isocyanate (0.25 g, 1.82 mmol) and 1-amino adamantane (0.27 g, 1.82 mmol), according to the general procedure. It was a white solid; yield—79% (0.41 g). ^1^H NMR (DMSO-*d*_6_, 500 MHz): δ 8.26 (s, 1H), 7.35–7.32 (m, 2H), 7.04–6.99 (m, 2H), 5.82 (s, 1H), 2.01 (s, 3H), 1.96–1.89 (m, 6H), 1.62 (s, 6H). ^13^C NMR (DMSO-*d*_6_, 125 MHz): δ 156.7 (d, ^1^J_C,F_ = 237.5 Hz), 154.0, 137.0, 118.8 (d, ^3^J_C,F_ = 7.2 Hz), 115.0 (d, ^2^J_C,F_ = 22.8 Hz), 49.8, 41.7, 36.1, 28.9. IR (KBr): ῡ = 3342.7, 2906.5, 2851.6, 1646.6, 1508.2, 1208.7, 828.2. LC-MS for C_17_H_21_FN_2_O (288.36): *m*/*z* = 289.33 [M + H]^+^.

##### Synthesis of 1-((3R,5S,7R)-3,5-dimethyladamantan-1-yl)-3-(4-fluorophenyl)urea (**3q**)

Compound (**3q**) was synthesized from 4-fluoro phenyl isocyanate (0.25g, 1.82 mmol) and 1-amino-3,5-dimethyladamantane (0.32 g, 1.82 mmol), according to the general procedure. It was a white solid; yield—76% (0.43 g). ^1^H NMR (DMSO-*d*_6_, 500 MHz): δ 8.25 (s, 1H), 7.36–7.32 (m, 2H), 7.04–6.99 (m, 2H), 5.84 (s, 1H), 2.08–2.06 (m, 1H), 1.75 (d, *J* = 2.9 Hz, 2H), 1.57 (s, 4H), 1.32 (d, *J* = 12.6 Hz, 2H), 1.24 (d, *J* = 12.6 Hz, 2H), 1.10 (s, 2H), 0.81 (s, 6H). ^13^C NMR (DMSO-*d*_6_, 125 MHz): δ 156.7 (d, ^1^J_C,F_ = 237.5 Hz), 154.1, 137.0, 118.8 (d, ^3^J_C,F_ = 7.2 Hz), 115.0 (d, ^2^J_C,F_ = 21.2 Hz), 51.5, 50.3, 47.8, 42.3, 40.2, 31.9, 30.1, 29.6. IR (KBr): ῡ = 3305.8, 2947.6, 2894.7, 1648.7, 1553.8, 1212.9, 830.2. LC-MS for C_19_H_25_FN_2_O (316.41): *m*/*z* = 317.35 [M + H]^+^.

### 3.3. Antimicrobial Studies

Samples were prepared in DMSO and water to a final testing concentration of 32 μg/mL or 20 μM (unless otherwise indicated in the datasheet), in 384-well, non-binding surface plate (NBS) for each bacterial/fungal strain, in duplicates (*n* = 2); the final DMSO concentration was kept to a maximum of 1% DMSO [29,30,31,32,33]. All sample preparations for the antimicrobial studies were done using liquid handling robots.

#### 3.3.1. Antimicrobial Assay

Primary antimicrobial screening study, by whole cell growth inhibition assays, were conducted using the compounds (**3a**–**q**) at a single concentration, in duplicates (*n* = 2). The inhibition of growth was measured against five bacteria—Escherichia coli (*E. coli*) ATCC 25922, Klebsiella pneumonia (*K. pneumoniae*) ATCC 700603, Acinetobacter baumannii (*A. Baumannii*) ATCC 19606, Pseudomonas aeruginosa (*P. aeruginosa*) ATCC 27853) and Staphylococcus aureus (*S. aureus*) ATCC 43300, and two fungi—*Candida albicans* ATCC 90028 and *Cryptococcus neoformans* ATCC 208821 [34].

##### Procedure

All bacteria were cultured in Cation-adjusted Mueller Hinton broth (CAMHB) at 37 °C, overnight. A sample of each culture was then diluted 40-fold in fresh broth and incubated at 37 °C for 1.5–3 h. The resultant mid-log phase cultures were diluted (CFU/mL measured by OD600) and then added to each well of the compound containing plates, giving a cell density of 5 × 10^5^ CFU/mL and a total volume of 50 μL. All plates were covered and incubated at 37 °C for 18 h without shaking.

##### Analysis

Inhibition of bacterial growth was determined by measuring absorbance at 600 nm (OD600), using a Tecan M1000 Pro monochromator plate reader. The percentage of growth inhibition was calculated for each well, using negative control (media only) and positive control (bacteria without inhibitors) on the same plate as references. The significance of the inhibition values was determined by modified Z-scores, calculated using the median and median absolute deviation (MAD) of the samples (no controls) on the same plate. Samples with inhibition value above 80% and a Z-Score above 2.5 for either replicate (*n* = 2 on different plates) were classed as actives. Samples with inhibition values between 50–80% and Z-Score above 2.5 for either replicate (*n* = 2 on different plates) were classed as partial actives.

#### 3.3.2. Antifungal Assay

##### Procedure

Fungi strains were cultured for 3 days on Yeast Extract–Peptone–Dextrose (YPD) agar at 30 °C. A yeast suspension of 1 × 10^6^ to 5 × 10^6^ CFU/mL (as determined by OD530) was prepared from five colonies. The suspension was subsequently diluted and added to each well of the compound-containing plates, giving a final cell density of the fungi suspension of 2.5 × 10^3^ CFU/mL and a total volume of 50 μL. All plates were covered and incubated at 35 °C for 24 h without shaking.

##### Analysis

Growth inhibition of *C. albicans* was determined by measuring absorbance at 530 nm (OD530), while the growth inhibition of *C. neoformans* was determined measuring the difference in absorbance between 600 and 570 nm (OD600–570), after the addition of resazurin (0.001% final concentration) and incubation at 35 °C for additional 2 h. The absorbance was measured using a Biotek Synergy HTX plate reader. The percentage of growth inhibition was calculated for each well, using a negative control (media only) and a positive control (fungi without inhibitors) on the same plate. The significance of the inhibition values was determined by the modified Z-scores, calculated using the median and the MAD of the samples (no controls) on the same plate. Samples with inhibition values above 80% and a Z-Score above 2.5 for either replicates (*n* = 2 on different plates) were classed as actives. Samples with inhibition values between 50–80% and Z-Score above 2.5 for either replicates (*n* = 2 on different plates) were classed as partial actives.

### 3.4. Docking Simulations

Molecular docking was used to clarify the binding mode of the compounds to provide straight forward information for further structural optimization. The crystal structure of *A. baumannii* PBP1a in complex with penicillin G (PDB ID 3UDI, 2.6 Å X-ray resolution) was extracted from the Brookhaven Protein Database (PDB http://www.rcsb.org/pdb). The proteins were prepared for docking by adding polar hydrogen atom with Gasteiger–Huckel charges and the water molecules were removed. The 3D structure of the ligands was generated by the SKETCH module implemented in the SYBYL program (Tripos Inc., St. Louis, USA) and its energy-minimized confirmation was obtained with the help of the Tripos force field using Gasteiger–Huckel [35] charges. Molecular docking was performed with the Surflex-Dock program, which was interfaced with Sybyl-X 2.0 [36], and other miscellaneous parameters were assigned with the default values given by the software.

## 4. Conclusions

In conclusion, we synthesized seventeen new urea derivatives (**3a**–**q**) with a simple one-step method in short reaction time (1 h) and with good yields (76–83%). The structures of all synthesized compounds were confirmed by IR, ^1^H NMR, ^13^C NMR, and mass spectroscopic techniques. The newly synthesized compounds were evaluated for their antimicrobial activity. Compounds **3c**, **3e**, **3f**, **3i**, **3j**, **3l**, and **3n** exhibited moderate to excellent growth inhibition against the *A. baumannii* bacterial strain. Among these seven compounds, compound **3l** displayed the highest growth inhibition towards the Gram-negative bacteria, *A. baumannii* (94.5%). Therefore, this 1-adamantyl urea could be considered as a promising antimicrobial lead and could form the structural backbone for further design and synthesis of other urea derivatives to be developed and screened. Furthermore, molecular docking studies of all synthesized compounds were carried out and good docking score towards *A. baumannii* were observed for all reported urea compounds.

## Figures and Tables

**Table 1:** Antimicrobial activity of compounds (**3a**–**q**) with the concentration set at 32 μg/mL in DMSO.

Compound (#)	Percentage of Inhibition of Growth ^[a]^						
	Antibacterial Activity					Antifungal Activity	
	Gram-Positive	Gram-Negative Bacteria					
	*Staphylococcus aureus*	*Escherichia coli*	*Pseudomonas aeruginosa*	*Klebsiella pneumoniae*	*Acinetobacter baumannii*	*Candida albicans*	*Cryptococcus neoformans*
3a	−0.1 ± 2.26	4 ± 0.98	−20.65 ± 3.18	−0.75 ± 2.75	−88.85 ± 22.82	**17.45 ± 19.44**	**30.65 ± 2.05**
3b	−29.7 5± 6.29	8.15 ± 2.61	**11.15 ± 15.48**	−7.4 ± 0.70	−58.1 ± 21.7	−1.4 ± 0.98	10.6 ± 12.58
3c	7.6 ± 1.55	3.3 ± 0.14	−2.34 ± 0.05	**29.35 ± 0.77**	20.1 ± 3.81	5.75 ± 2.19	−4.4 ± 0.42
3d	7.25 ± 0.49	5.35 ± 0.91	10.95 ± 15.62	17.55 ± 0.77	11 ± 31.39	−5.6 ± 0.00	2.2 ± 2.96
3e	0.45 ± 3.60	9.2 ± 0.00	−5.35 ± 18.17	−5.55 ± 6.15	**51.85 ± 12.94**	2.75 ± 1.34	−2.35 ± 7.56
3f	1.6 ± 0.28	3.55 ± 1.48	−12 ± 6.50	−4.9 ± 7.91	25.9 ± 27.86	3.15 ± 2.47	−3.2 ± 9.47
3g	−5.8 ± 4.38	2.2 ± 5.09	−3.65 ± 10.53	**21.25 ± 4.31**	−6.3 ± 33.79	−4.0 ± 3.67	−9.85 ± 2.75
3h	−0.9 ± 0.70	9.45 ± 3.18	2.3 ± 15.98	−3.65 ± 1.34	13.75 ± 46.31	2.2 ± 1.97	−4.45 ± 6.01
3i	12.4 ± 4.24	**10.15 ± 0.63**	−4.6 ± 15.13	11.4 ± 8.62	25.15 ± 31.59	6.25 ± 0.49	−6.2 ± 14.00
3j	−11.05 ± 1.48	−1.65 ± 4.87	−17.45 ± 6.29	0.95 ± 3.18	**49.35 ± 49.69**	**13.4 ± 3.81**	−0.8 ± 19.37
3k	**24.9 ± 1.13**	−0.5 ± 2.40	−8.5 ± 6.50	−11.8 ± 0.70	−97.4 ± 93.76	4.45 ± 4.17	**15.25 ± 1.76**
3l	13.25 ± 0.49	−1.2 ± 0.28	−16.5 ± 0.14	−16 ± 1.69	**94.5 ± 17.23**	3.05 ± 3.32	12.05 ± 3.88
3m	−23.5 ± 3.95	11.65 ± 0.07	−6.15 ± 22.41	−6.65 ± 1.20	−16.15 ± 8.27	0.35 ± 1.62	11.3 ± 7.91
3n	7.8 ± 1.69	7.9 ± 0.14	−3.25 ± 6.71	2.0 ± 1.13	**46.35 ± 16.33**	1.25 ± 3.88	−3.75 ± 1.06
3o	8.35 ± 0.07	2.25 ± 1.48	−5.4 ± 13.43	−21.5 ± 0.70	2.65 ± 33.44	0.00 ± 3.39	−2.6 ± 6.08
3p	2.15 ± 9.26	5.9 ± 2.26	−1.65 ± 19.86	−7.85 ± 2.61	14.6 ± 30.97	3.45 ± 1.20	3.4 ± 3.25
3q	7.55 ± 0.35	10.45 ± 0.07	−0.55 ± 16.05	−5.0 ± 4.24	14.9 ± 36.91	0.2 ± 3.25	−3.35 ± 4.73

^[a]^ Percentage growth inhibition at a concentration of 32 μg·mL^−1^ calculated on the basis of negative (media only) and positive controls (bacterial/fungal media without inhibitor, set to 0%). Variation ± 10%. Colistin [Minimum Inhibition Concentration (MIC) 0.125–0.25 μg/mL] and vancomycin (MIC 1 μg/mL) were used as positive bacterial inhibitor standards for Gram-negative and Gram-positive bacteria, respectively. Fluconazole was used as a positive fungal inhibitor standard for *C. albicans* (MIC 0.125 μg/mL) and *C. neoformans* (MIC 8 μg/mL). Highest percentiles of antibacterial/antifungal growth inhibition are highlighted in bold. Data are expressed as the mean ± SD. SD—Standard Deviation. Best inhibition results are indicated in **bold**.

**Table 2:** Surflex Docking Score (kcal/mol) of the urea derivatives.

Compounds	C Score ^a^	Crash Score ^b^	Polar Score ^c^	D Score ^d^	PMF Score ^e^	G Score ^f^	Chem Score ^g^
**3UDI_Ligand**	5.12	−1.73	5.34	−127.811	−36.763	−199.315	−26.957
**3a**	4.91	−0.99	0.43	−96.944	15.031	−156.919	−21.467
**3b**	4.98	−1.67	1.04	−108.502	−16.998	−206.677	−28.934
**3c**	4.86	−1.63	1.11	−106.679	−22.362	−177.149	−23.114
**3d**	4.75	−0.50	1.38	−72.791	21.318	−141.071	−19.363
**3e**	3.58	−1.22	0.00	−87.507	31.814	−143.787	−15.604
**3f**	4.73	−1.53	1.05	−108.037	−21.206	−206.699	−28.745
**3g**	4.17	−1.30	1.09	−102.700	−24.068	−171.270	−22.764
**3h**	2.91	−0.47	0.00	−79.382	−16.486	−124.882	−19.191
**3i**	3.44	−1.36	0.82	−94.042	−29.559	−161.576	−23.494
**3j**	4.96	−1.62	0.91	−105.444	−20.461	−208.403	−27.100
**3k**	4.37	−0.95	2.27	−95.562	−30.750	−146.048	−26.797
**3l**	3.61	−0.56	0.97	−86.268	−0.930	−140.976	−20.941
**3m**	5.19	−1.60	0.73	−101.453	41.298	−176.730	−19.940
**3n**	5.19	−1.94	0.08	−121.156	19.866	−212.017	−27.491
**3o**	4.43	−1.30	0.99	−99.796	−23.667	−166.866	−22.226
**3p**	3.48	−0.92	0.09	−85.783	17.463	−127.721	−18.822
**3q**	3.46	−0.66	0.00	−87.632	10.207	−129.574	−20.064

^a^ CScore (Consensus Score) integrates a number of popular scoring functions for ranking the affinity of ligands bound to the active site of a receptor and reports the output of the total score. ^b^ Crash-score revealing the inappropriate penetration into the binding site. Crash scores close to 0 are favorable. Negative numbers indicate penetration. ^c^ Polar indicating the contribution of the polar interactions to the total score. The polar score might be useful for excluding the docking results that make no hydrogen bonds. ^d^ D-score for charge and van der Waals interactions between the protein and the ligand. ^e^ PMF-score indicating the Helmholtz free energies of interactions for the protein–ligand atom pairs (Potential of Mean Force (PMF)). ^f^ G-score showing hydrogen bonding, complex (ligand–protein), and internal (ligand–ligand) energies. ^g^ Chem-score points for H-bonding, lipophilic contact, and rotational entropy, along with an intercept term.

## Supplementary Materials

Supplementary data [^1^H and ^13^C NMR data of all the compounds (**3a**–**q**)]. Supplementary data to this article can be found online at https://www.mdpi.com/2079-6382/8/4/178/s1.
