# Supplementary material for: New Urea Derivatives as Potential Antimicrobial Agents: Synthesis, Biological Evaluation, and Molecular Docking Studies
Source: Antibiotics (Basel). 2019 Oct 9;8(4):178. doi: 10.3390/antibiotics8040178 (PMC6963781; doi:10.3390/antibiotics8040178)

Supplementary Materials

New Urea Derivatives as Potential Antimicrobial Agents: Synthesis, Biological Evaluation, and Molecular Docking Studies

Mahadev Patil[a], Anurag Noonikara Poyil[b], Shrinivas D. Joshi[c], Shivaputra A. Patil[d], Siddappa A. Patil[a]*, and Alejandro Bugarin[e]*

[a]*Centre for Nano & Material Sciences, Jain University, Jain Global Campus, Bangalore 562112, Karnataka, India.*
[b]*Department of Chemistry and Biochemistry, University of Texas at Arlington, Arlington, TX 76019, USA*
[c]*Novel Drug Design and Discovery Laboratory, Department of Pharmaceutical Chemistry, S. E. T's College of Pharmacy, Sangolli Rayanna Nagar, Dharwad 580 002, Karnataka, India.*
[d]*Pharmaceutical Sciences Department, College of Pharmacy, Rosalind Franklin University of Medicine and Science, 3333 Green Bay Road, North Chicago, IL 60064, USA*
[e]*Department of Chemistry and Physics, Florida Gulf Coast University, Fort Myers, FL 33965, USA.*

Table of Contents

^{1}H and ^{13}C NMR Spectra S-2

*Corresponding authors

E-mail: abugarin@fgcu.edu (Dr. Alejandro Bugarin)

p.siddappa@jainuniversity.ac.in (Dr. Siddappa A. Patil)

^{1}H and ^{13}C NMR Spectra

^{1}H NMR (500 MHz, $CDCl_3$)

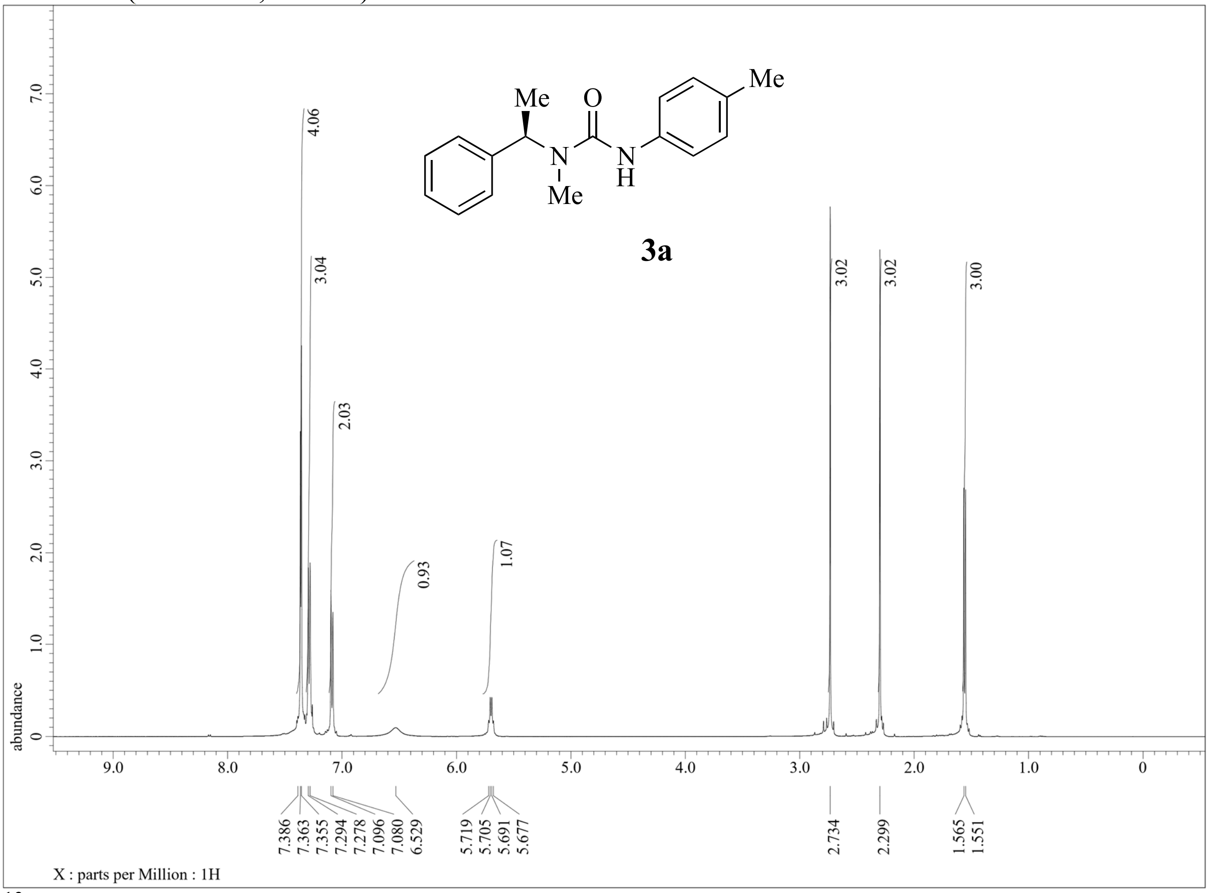

^{13}C NMR (125 MHz, $CDCl_3$)

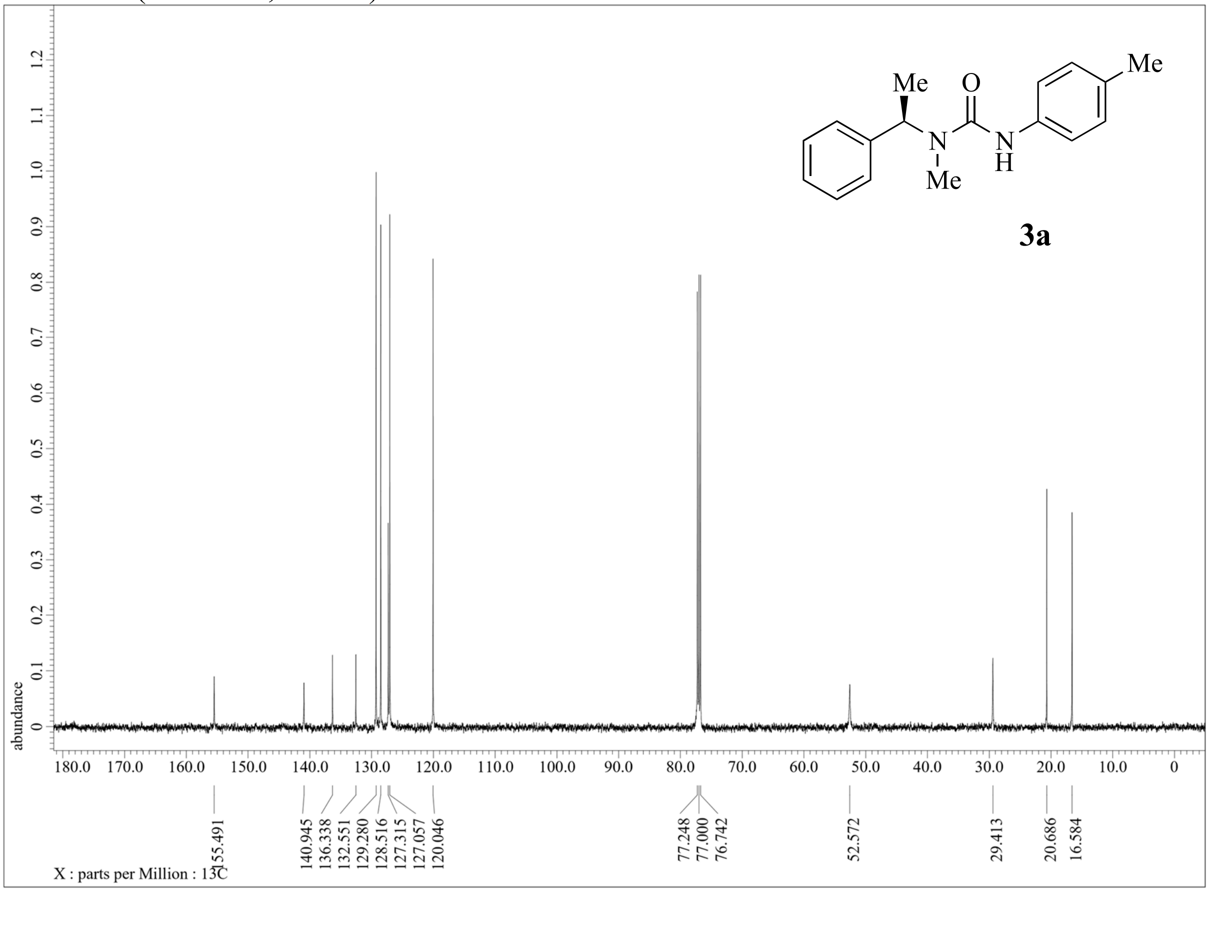

^{1}H NMR (500 MHz, DMSO-d_6)

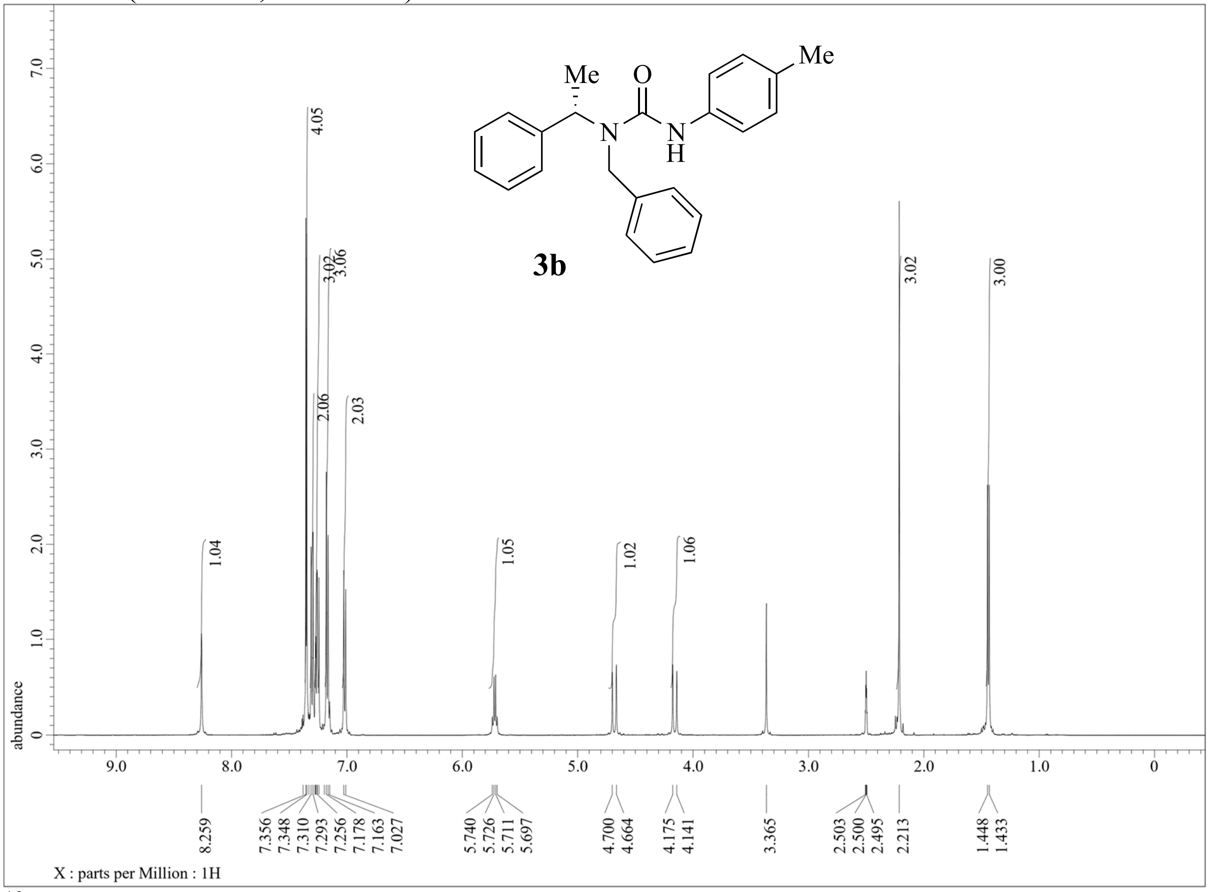

^{13}C NMR (125 MHz, DMSO-d_6)

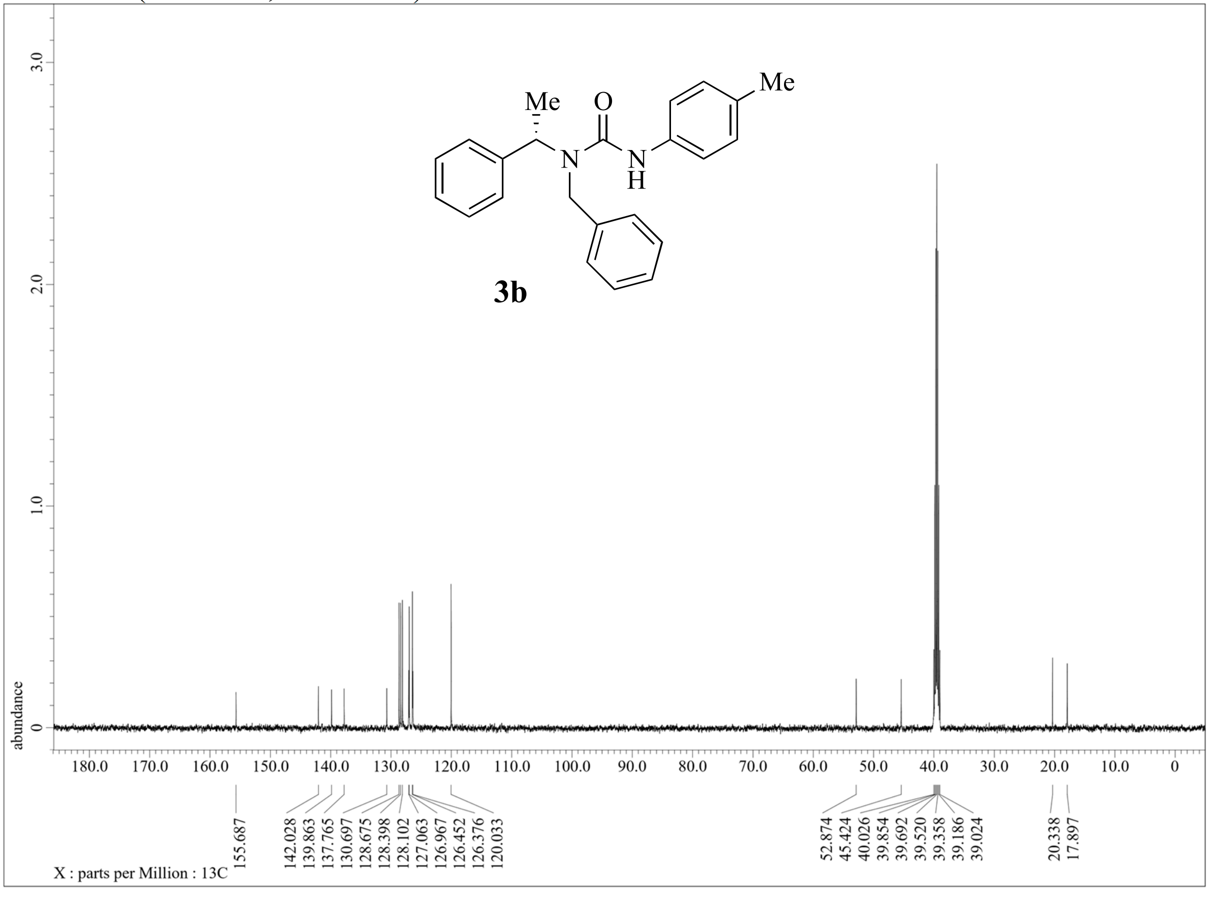

^{1}H NMR (500 MHz, DMSO-d_6)

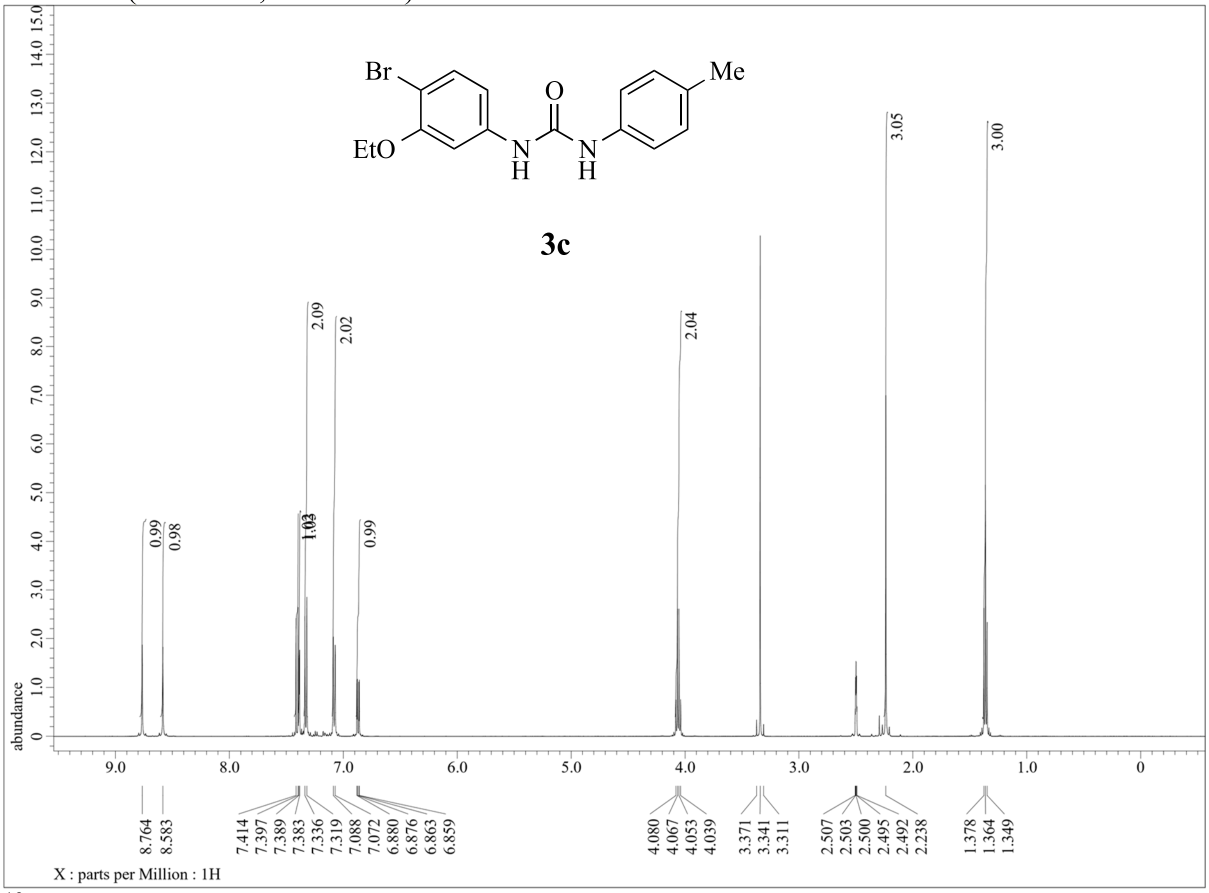

^{13}C NMR (125 MHz, DMSO-d_6)

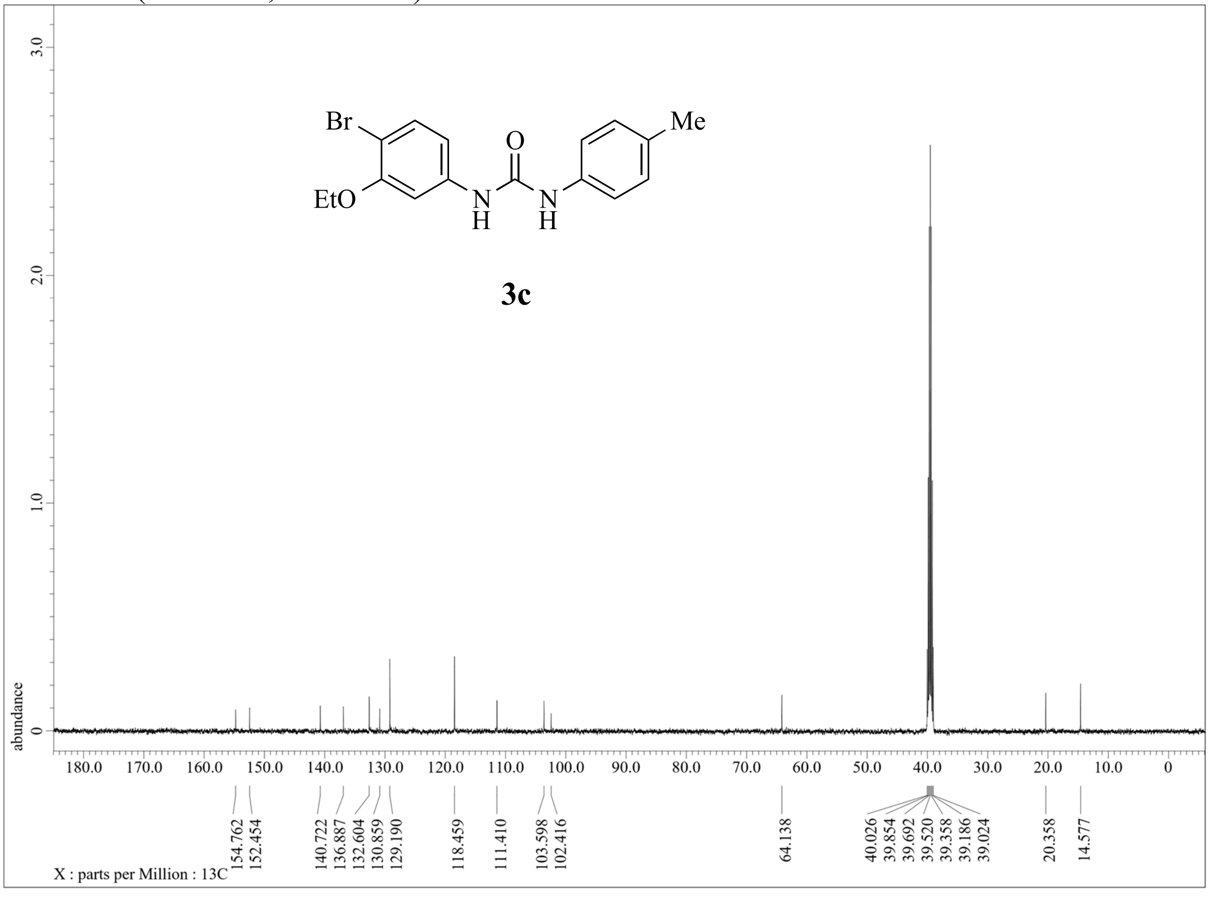

^{1}H NMR (500 MHz, DMSO-d_6)

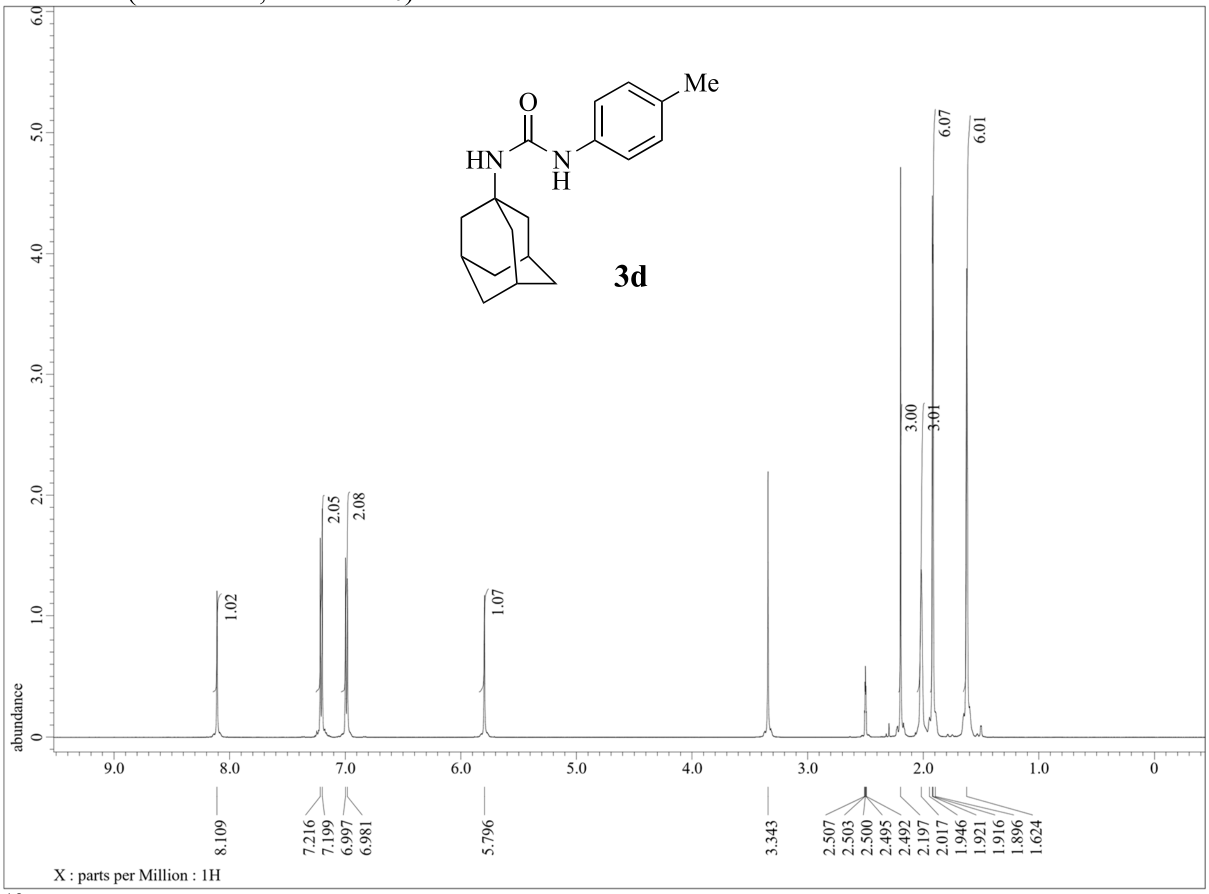

^{13}C NMR (125 MHz, DMSO-d_6)

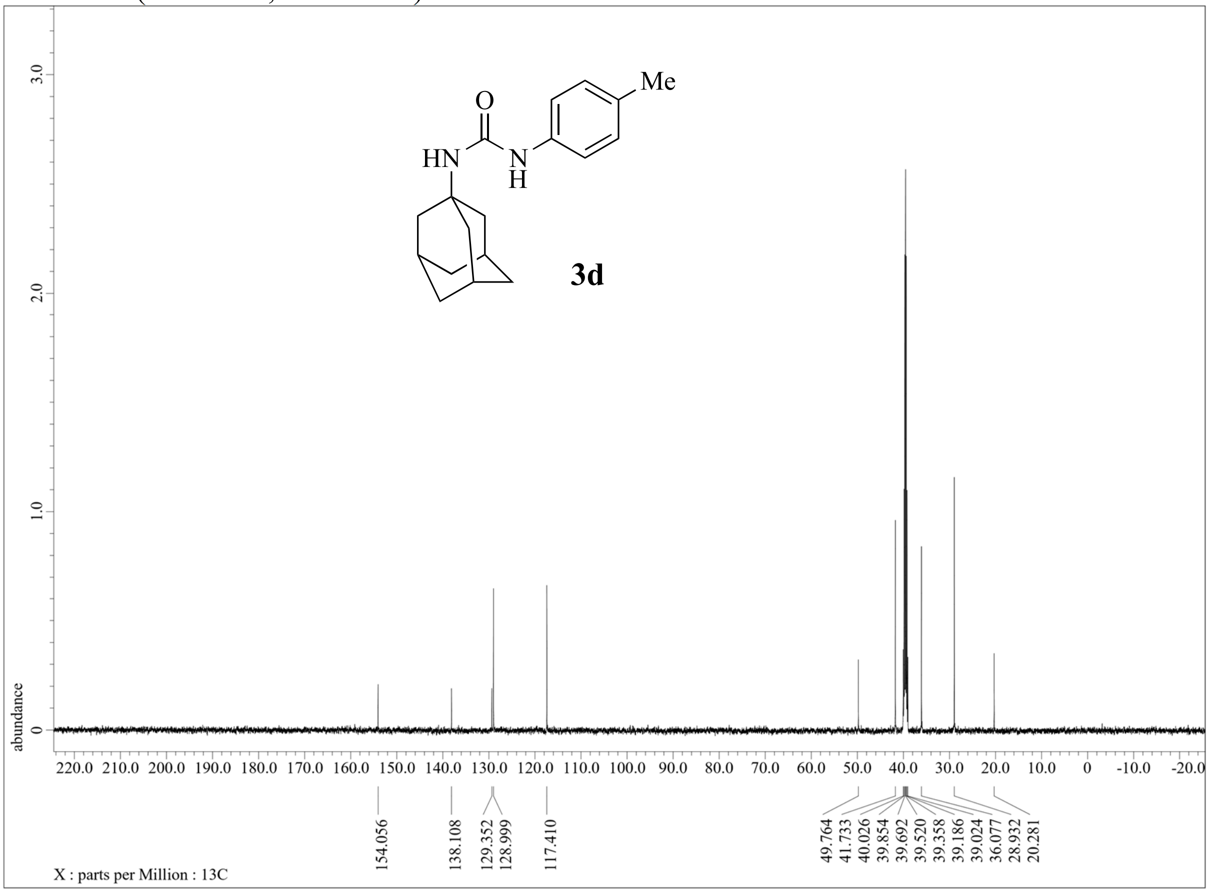

^{1}H NMR (500 MHz, DMSO-d_6)

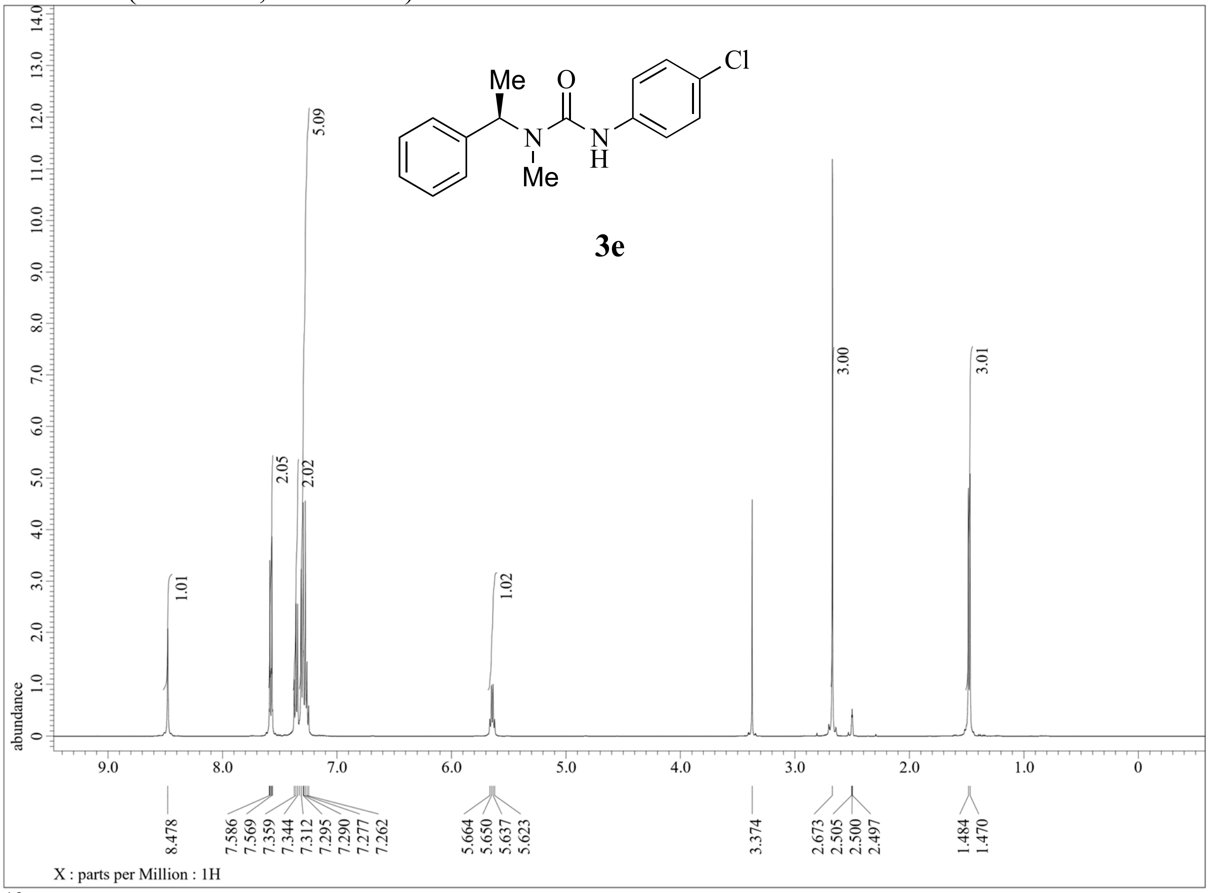

^{13}C NMR (125 MHz, DMSO-d_6)

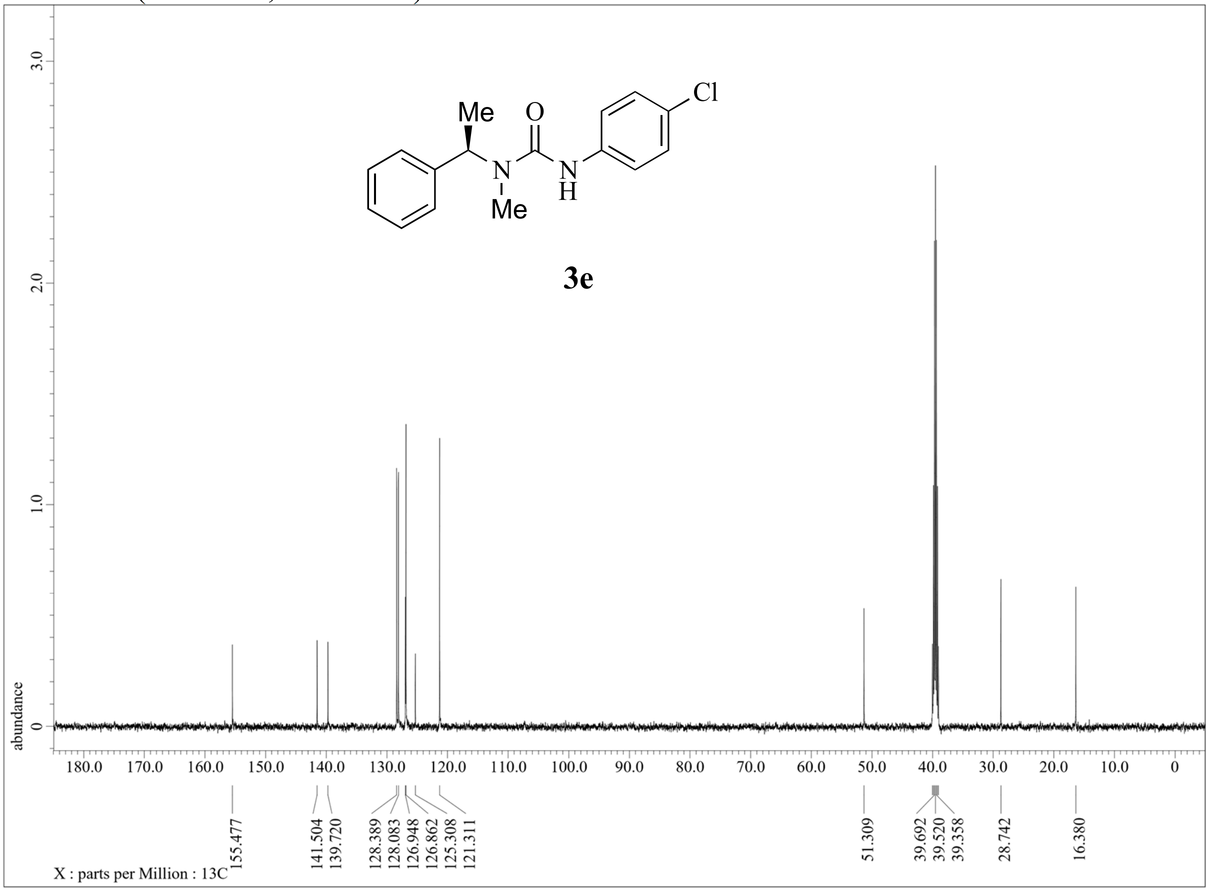

^{1}H NMR (500 MHz, DMSO-d_6)

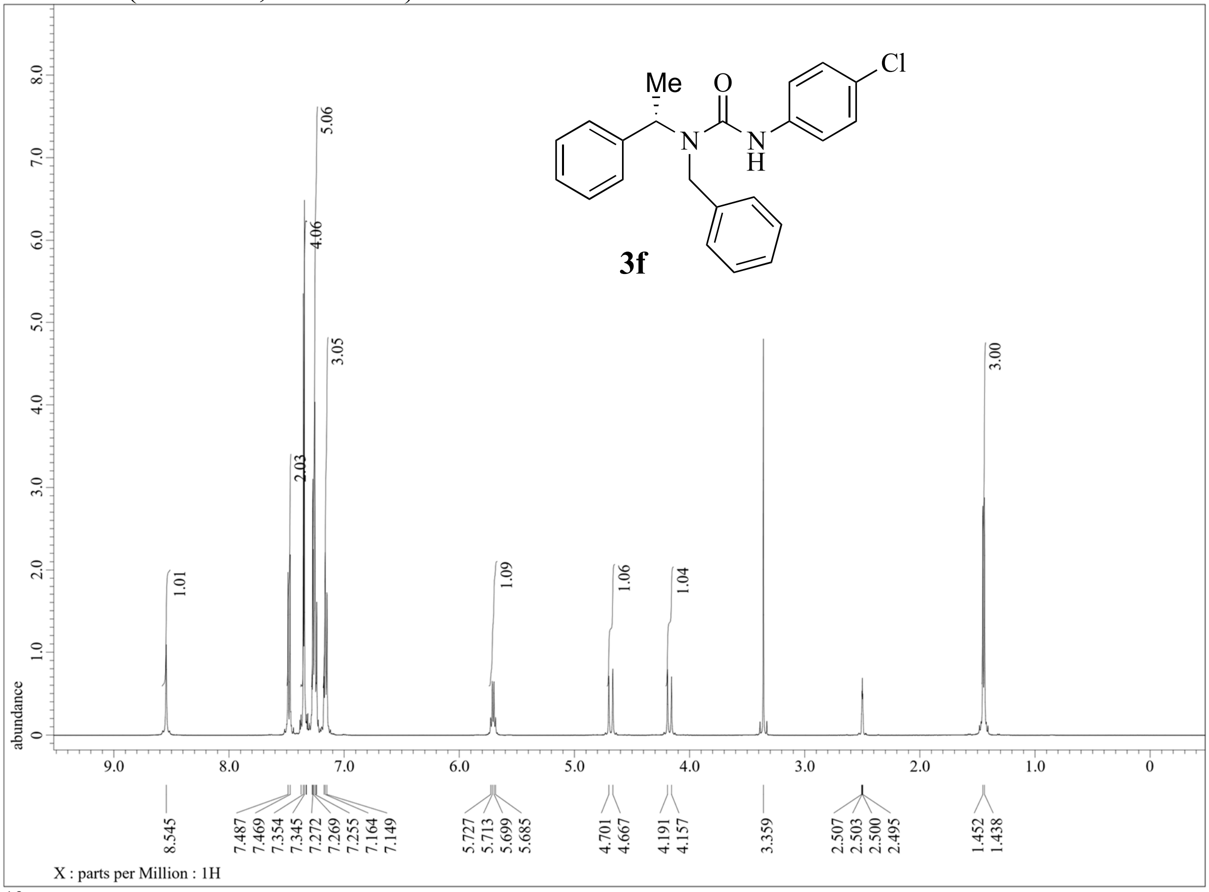

^{13}C NMR (125 MHz, DMSO-d_6)

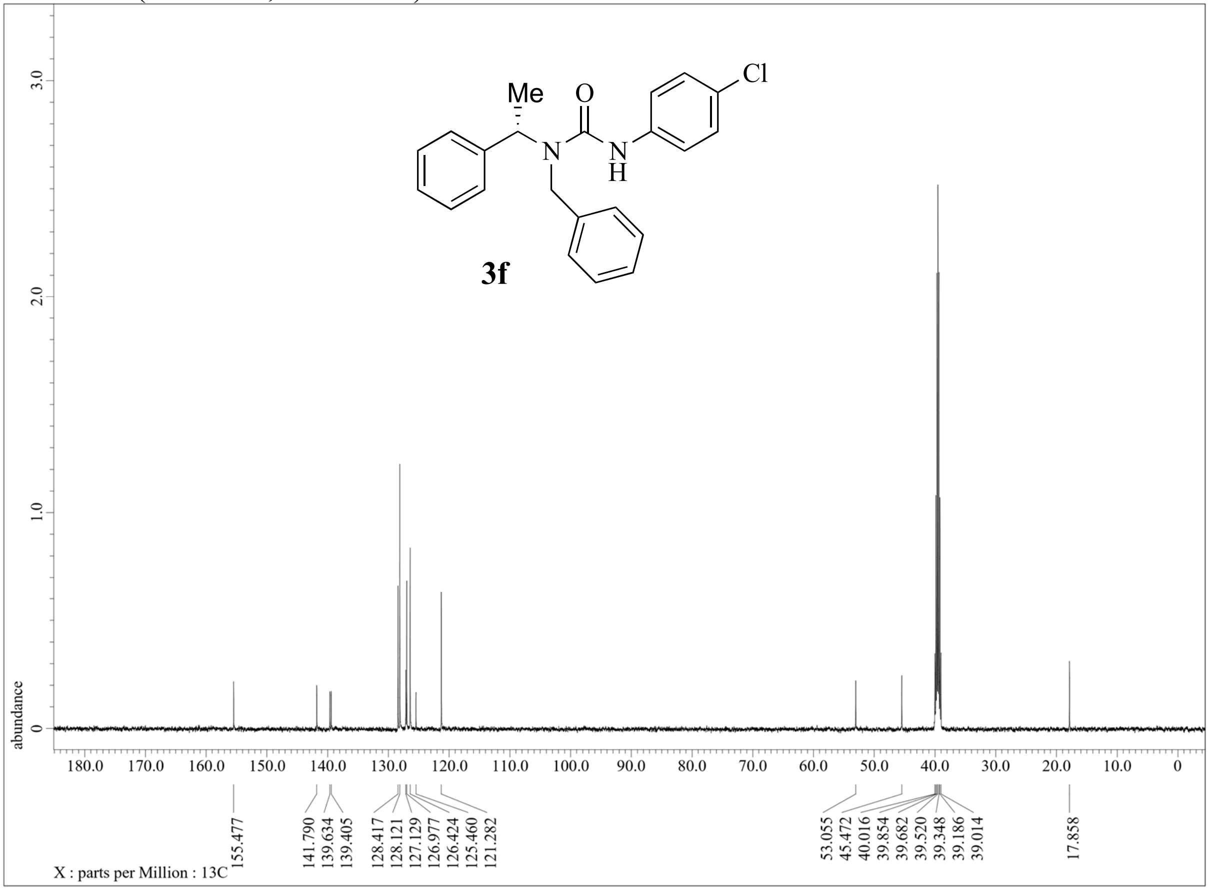

^{1}H NMR (500 MHz, DMSO-d_6)

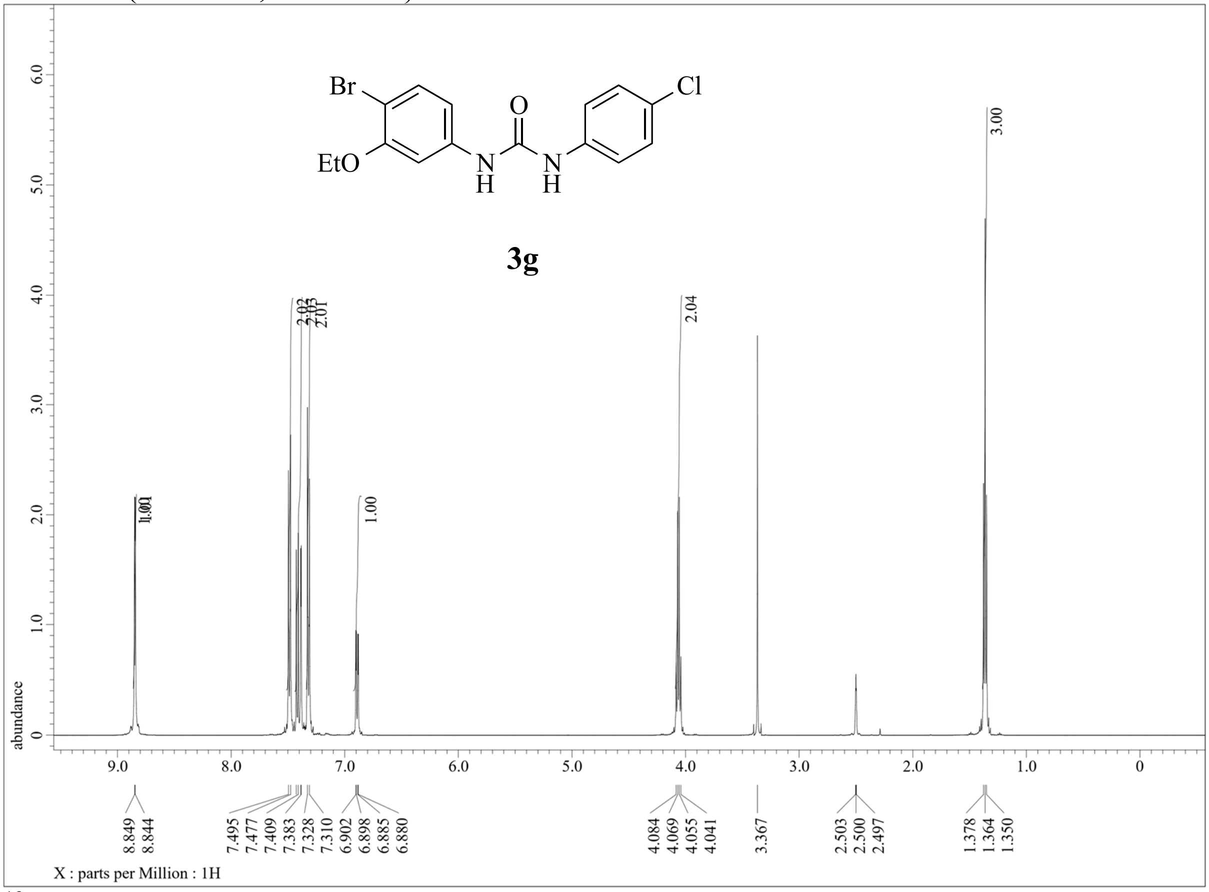

^{13}C NMR (125 MHz, DMSO-d_6)

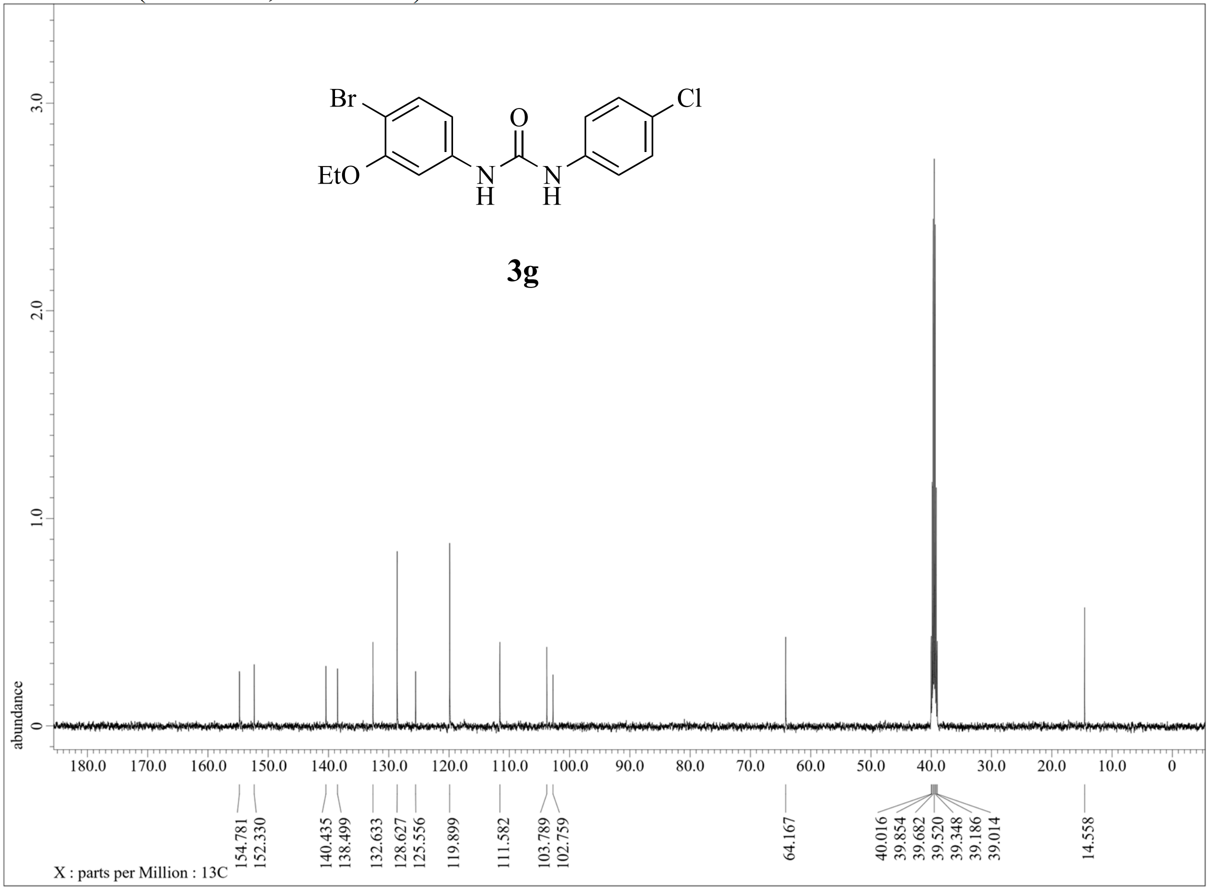

^{1}H NMR (500 MHz, DMSO-d_6)

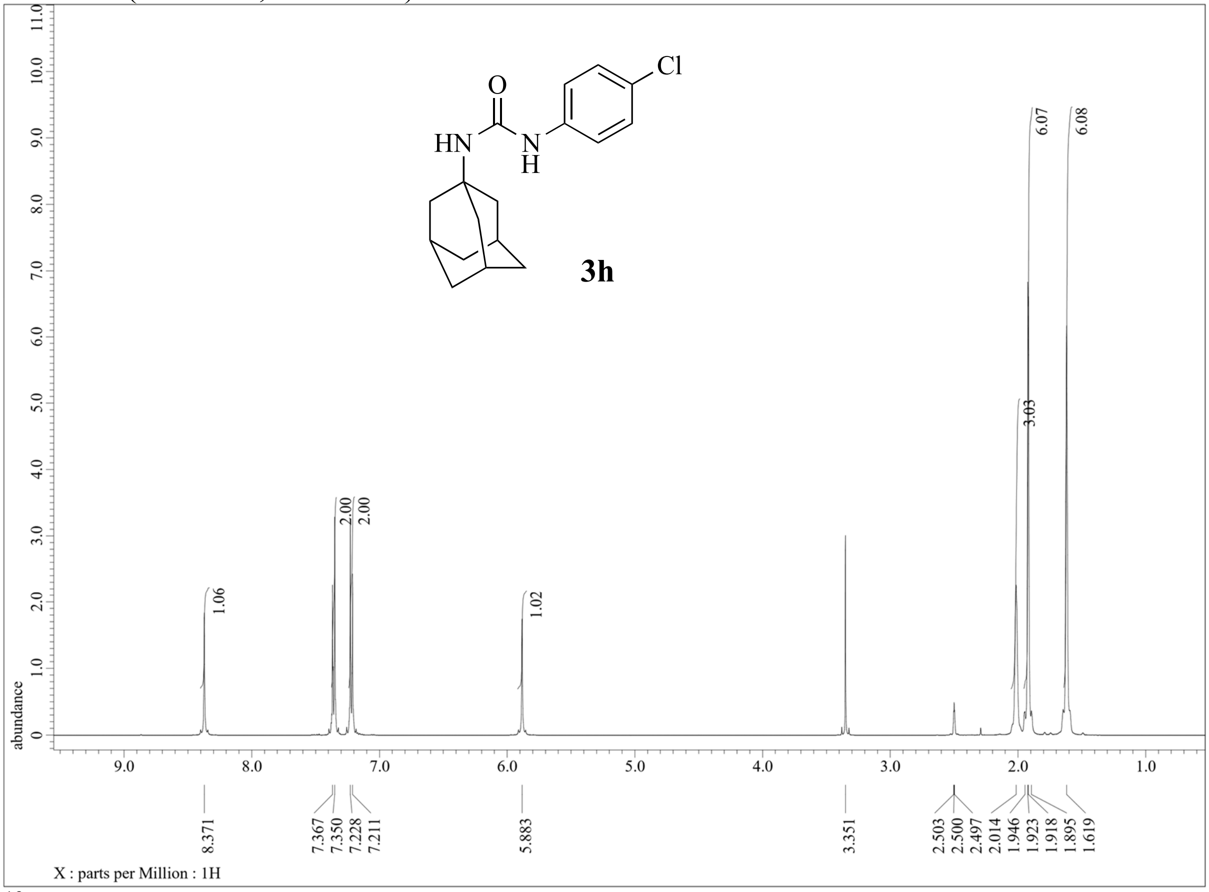

^{13}C NMR (125 MHz, DMSO-d_6)

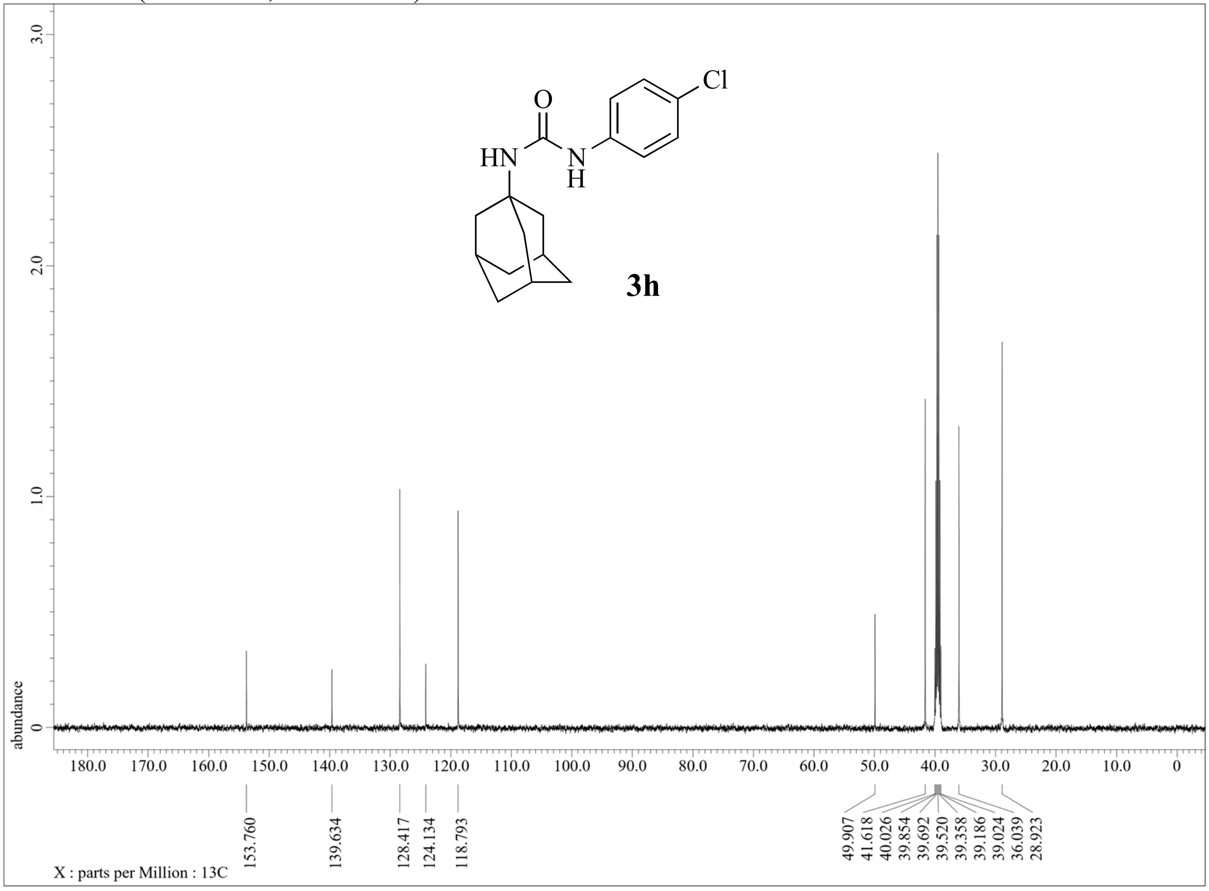

^{1}H NMR (500 MHz, DMSO-d_6)

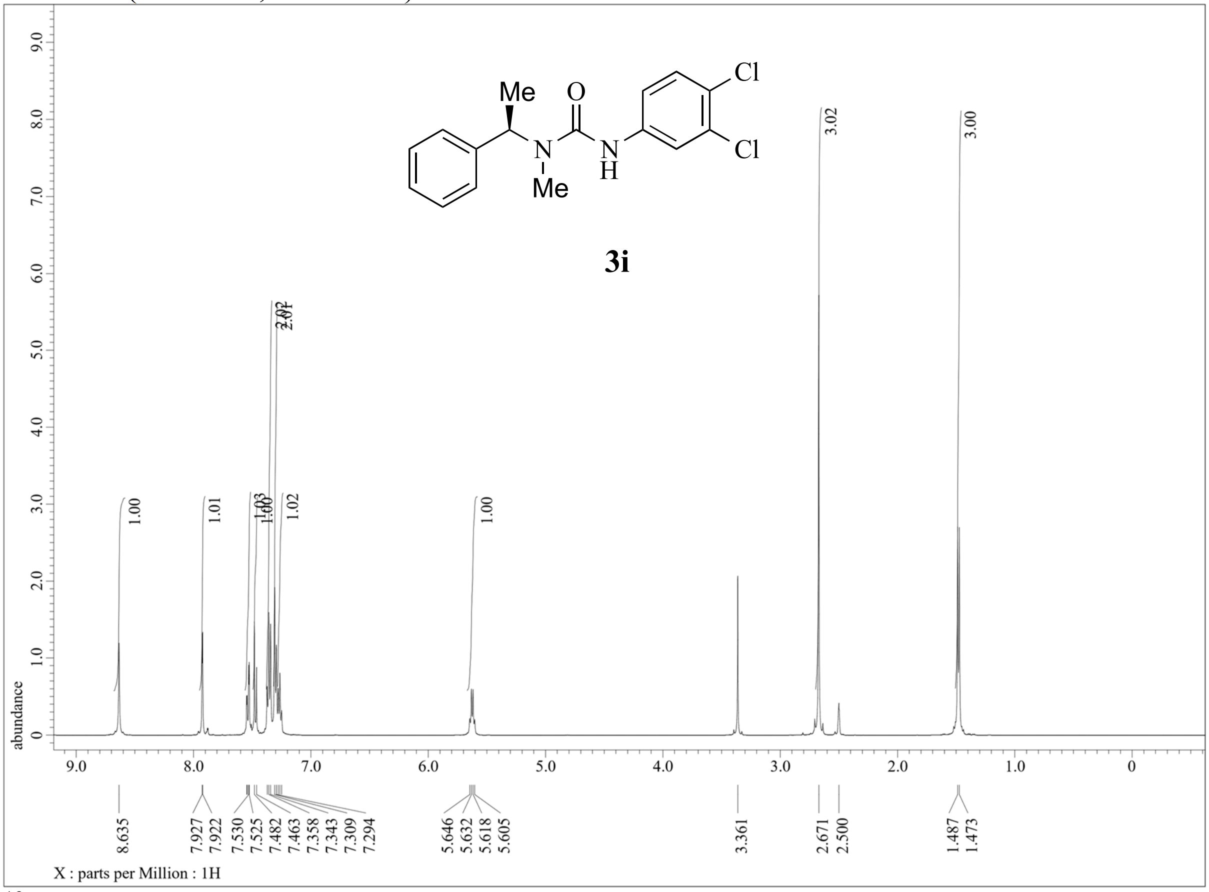

^{13}C NMR (125 MHz, DMSO-d_6)

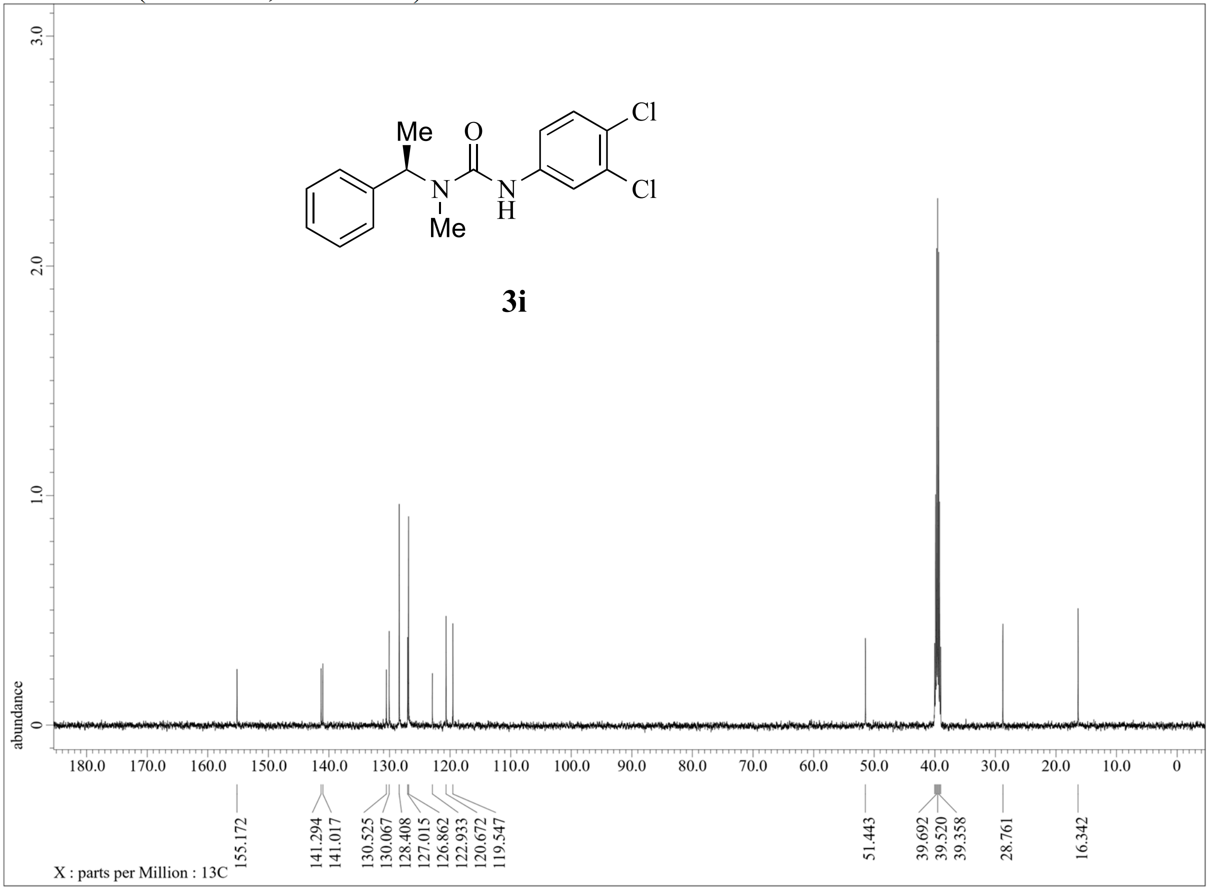

^{1}H NMR (500 MHz, DMSO-d_6)

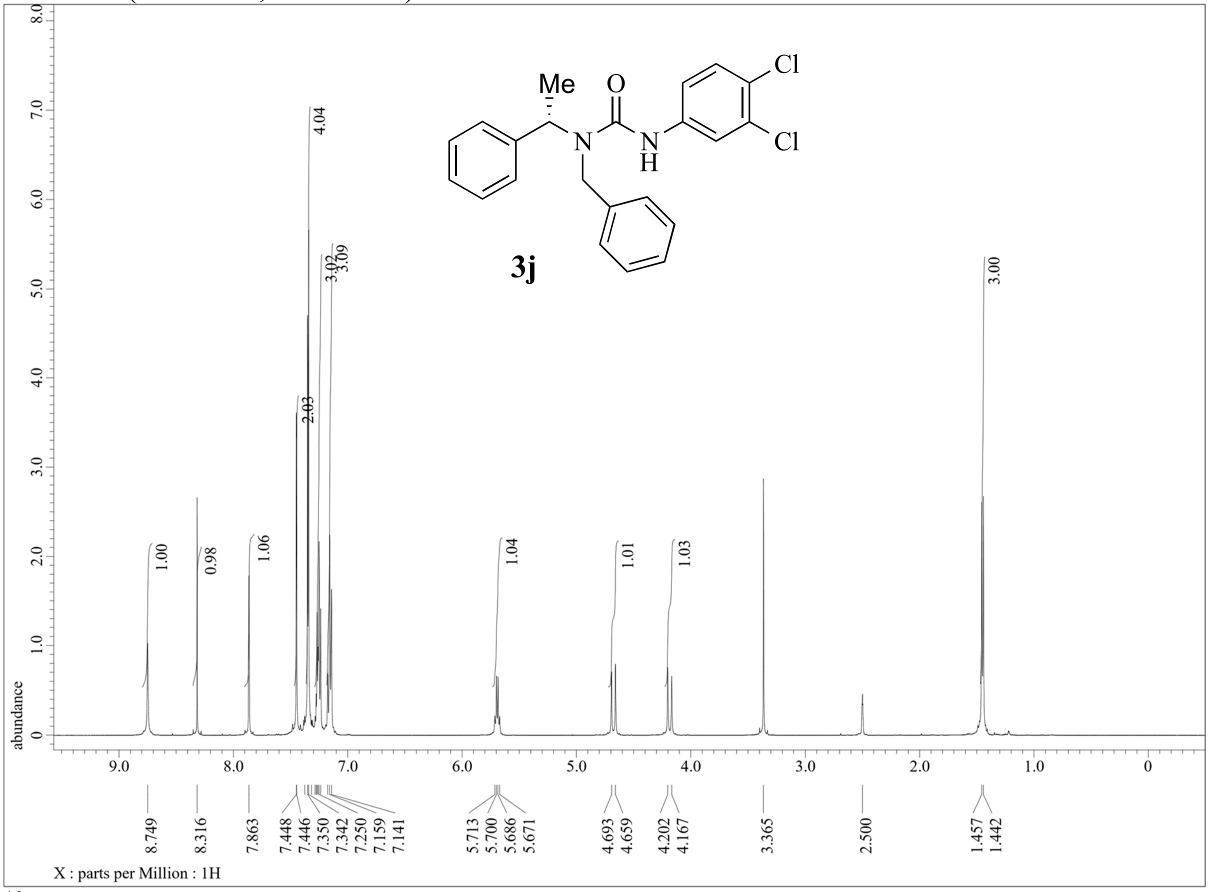

^{13}C NMR (125 MHz, DMSO-d_6)

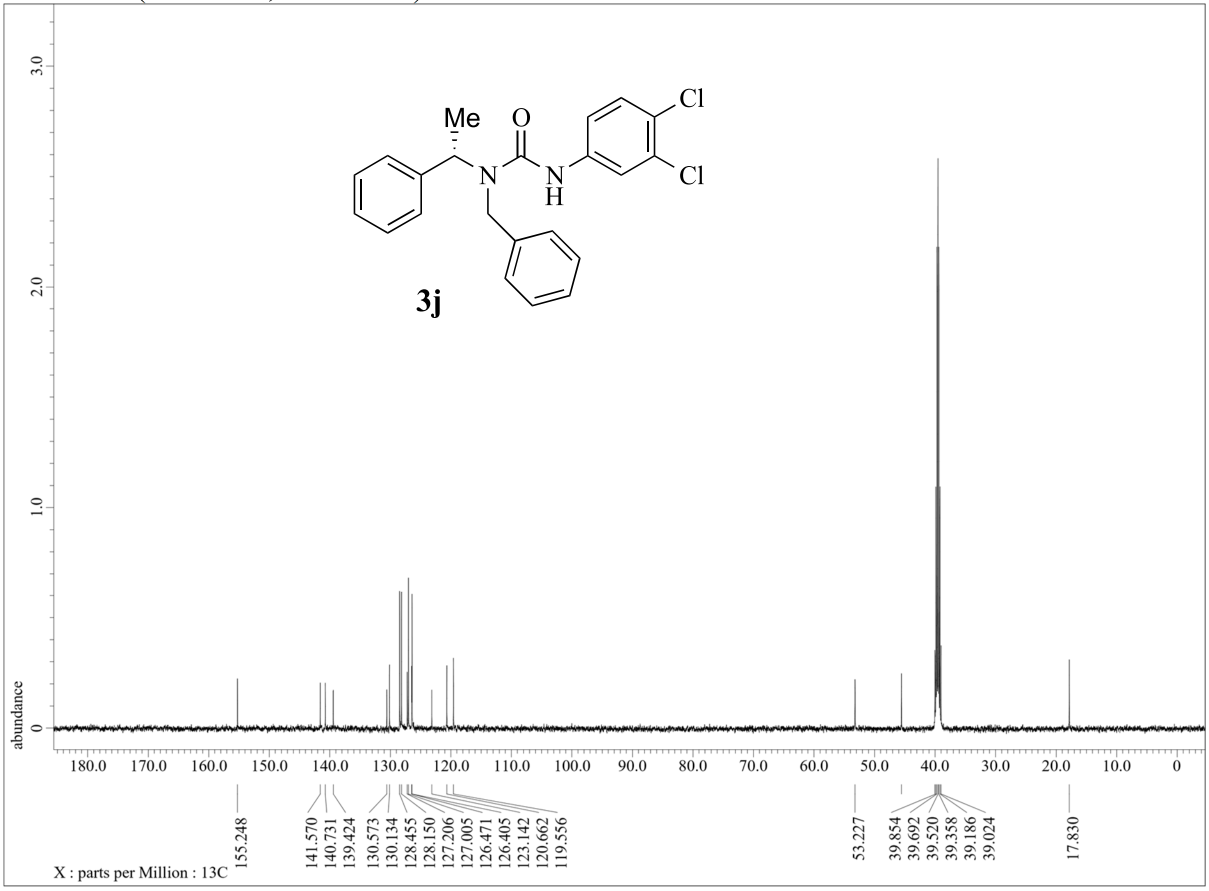

^{1}H NMR (500 MHz, DMSO-d_6)

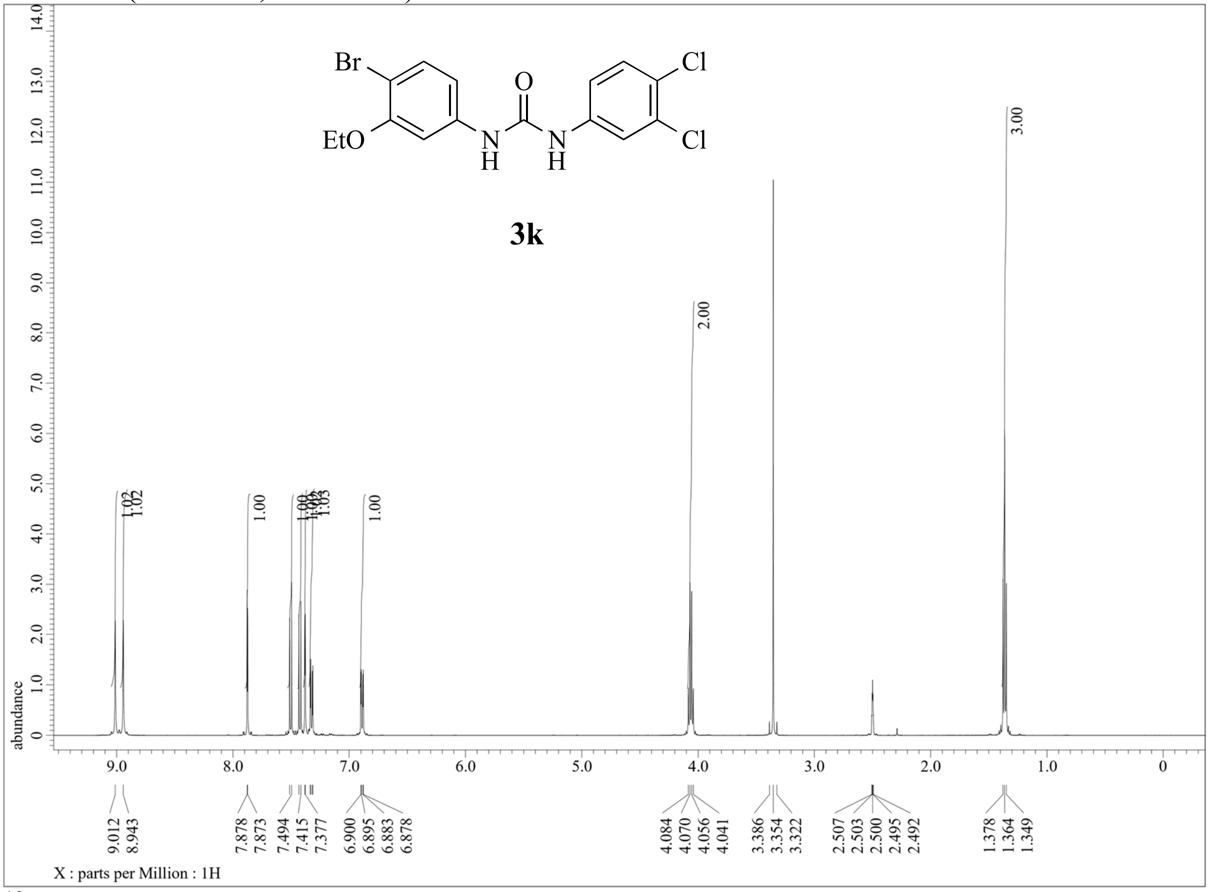

^{13}C NMR (125 MHz, DMSO-d_6)

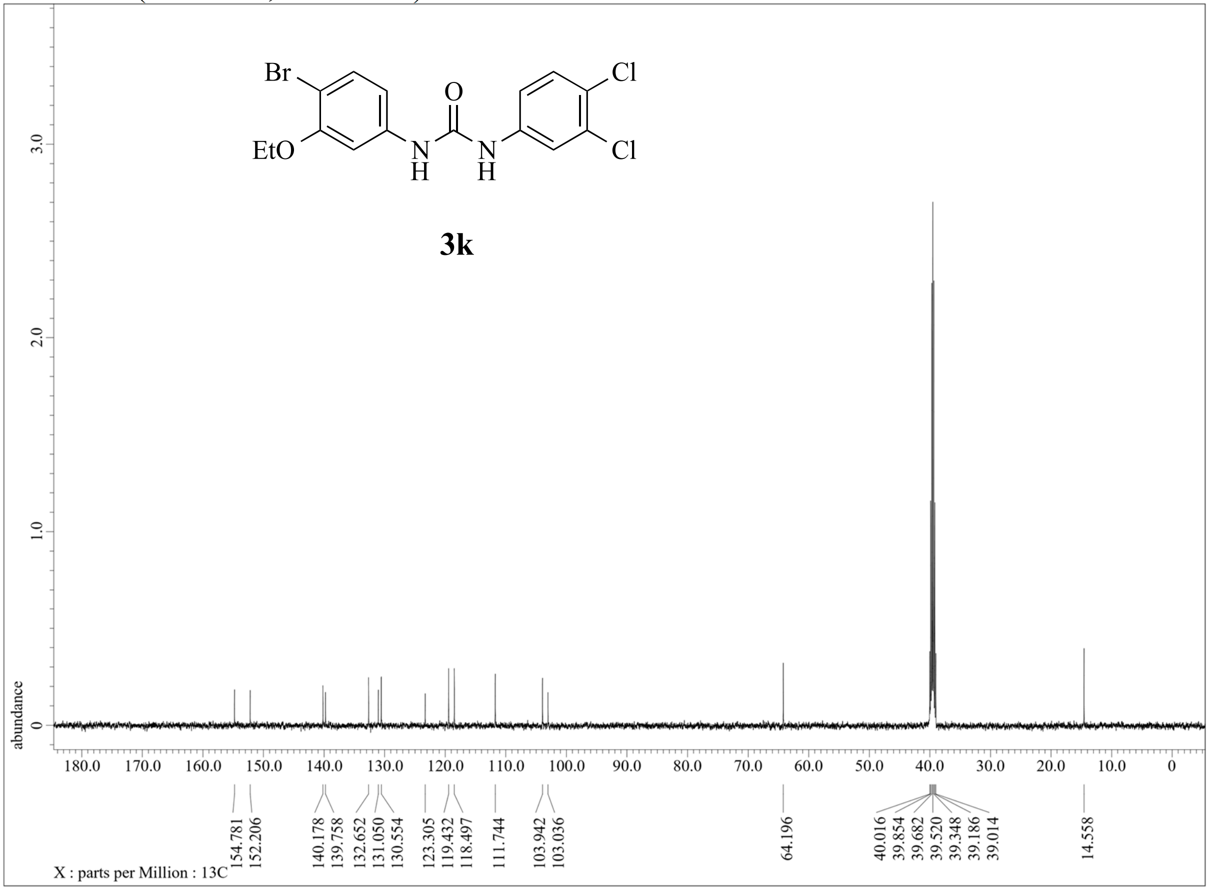

^{1}H NMR (500 MHz, DMSO-d_6)

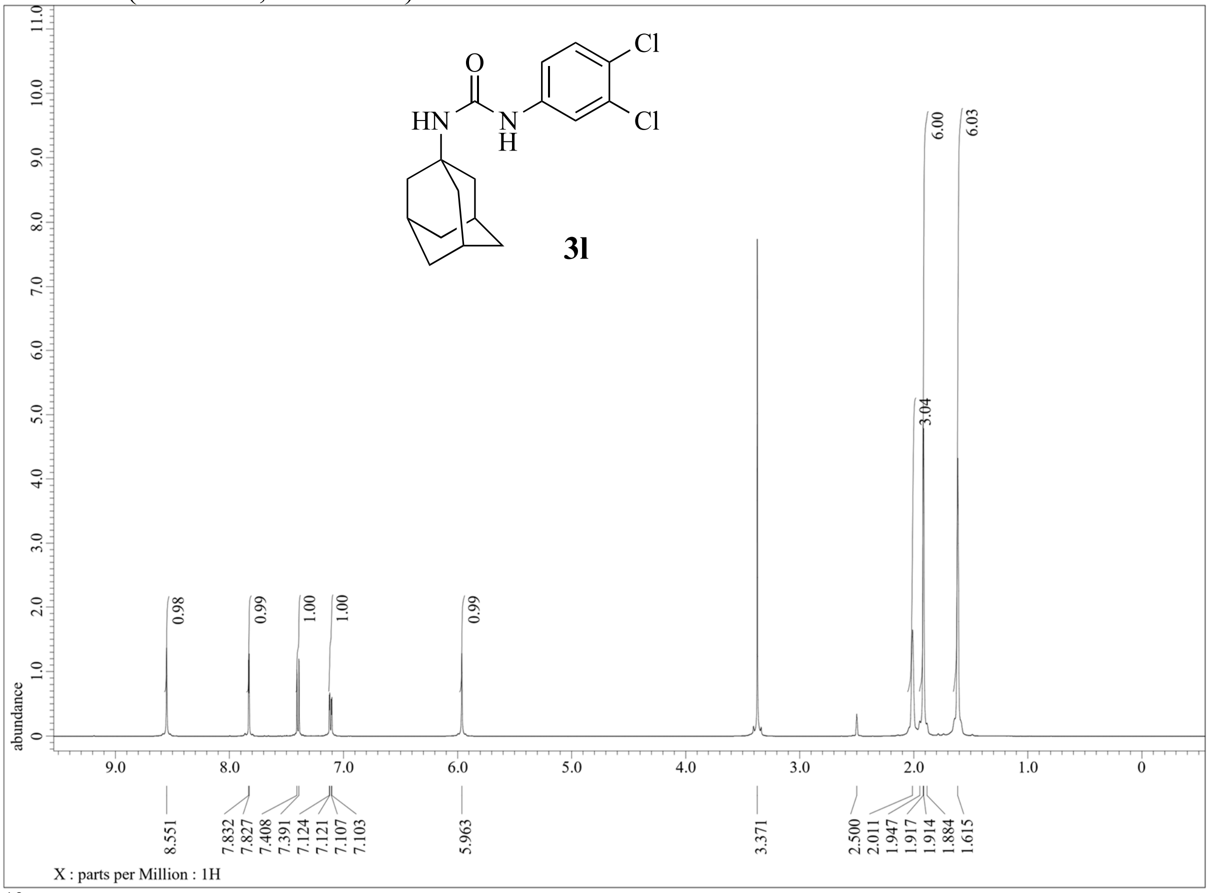

^{13}C NMR (125 MHz, DMSO-d_6)

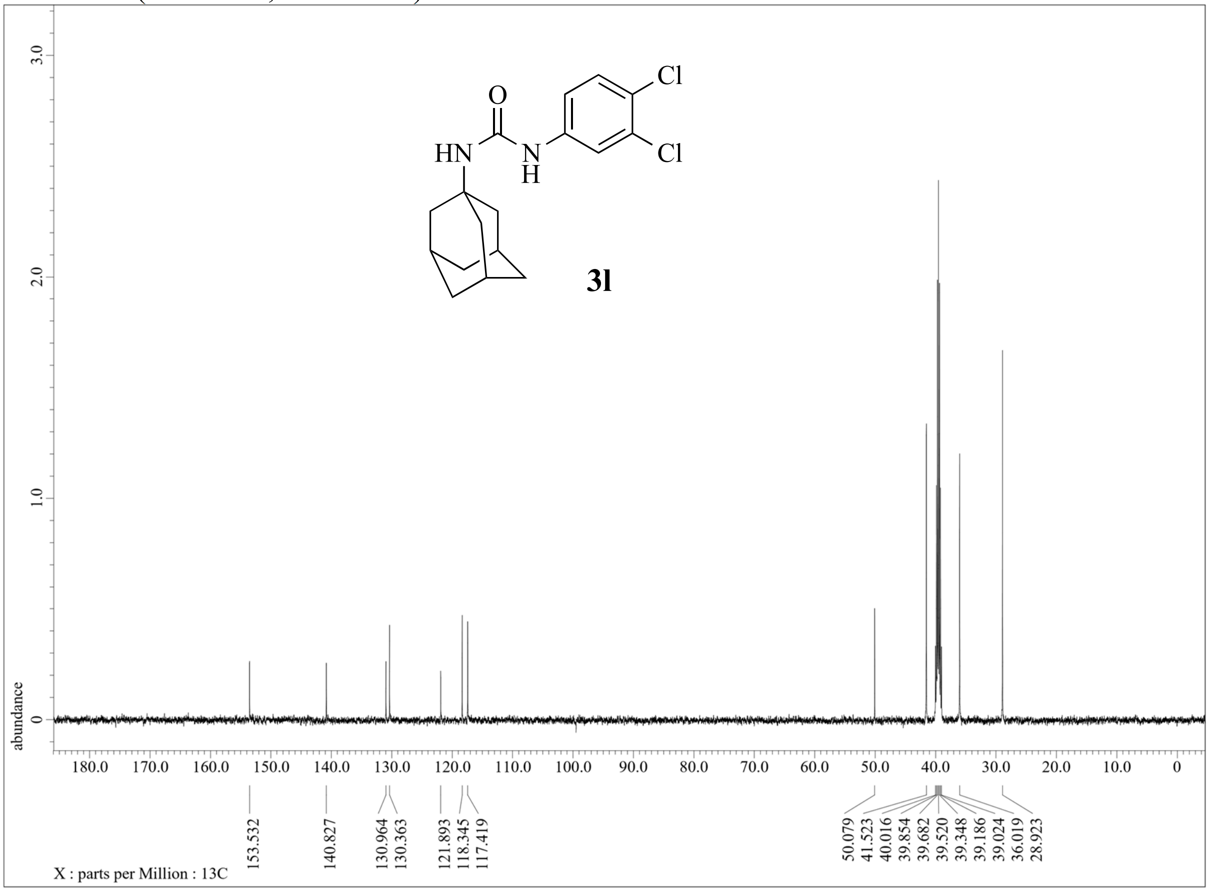

^{1}H NMR (500 MHz, DMSO-d_6)

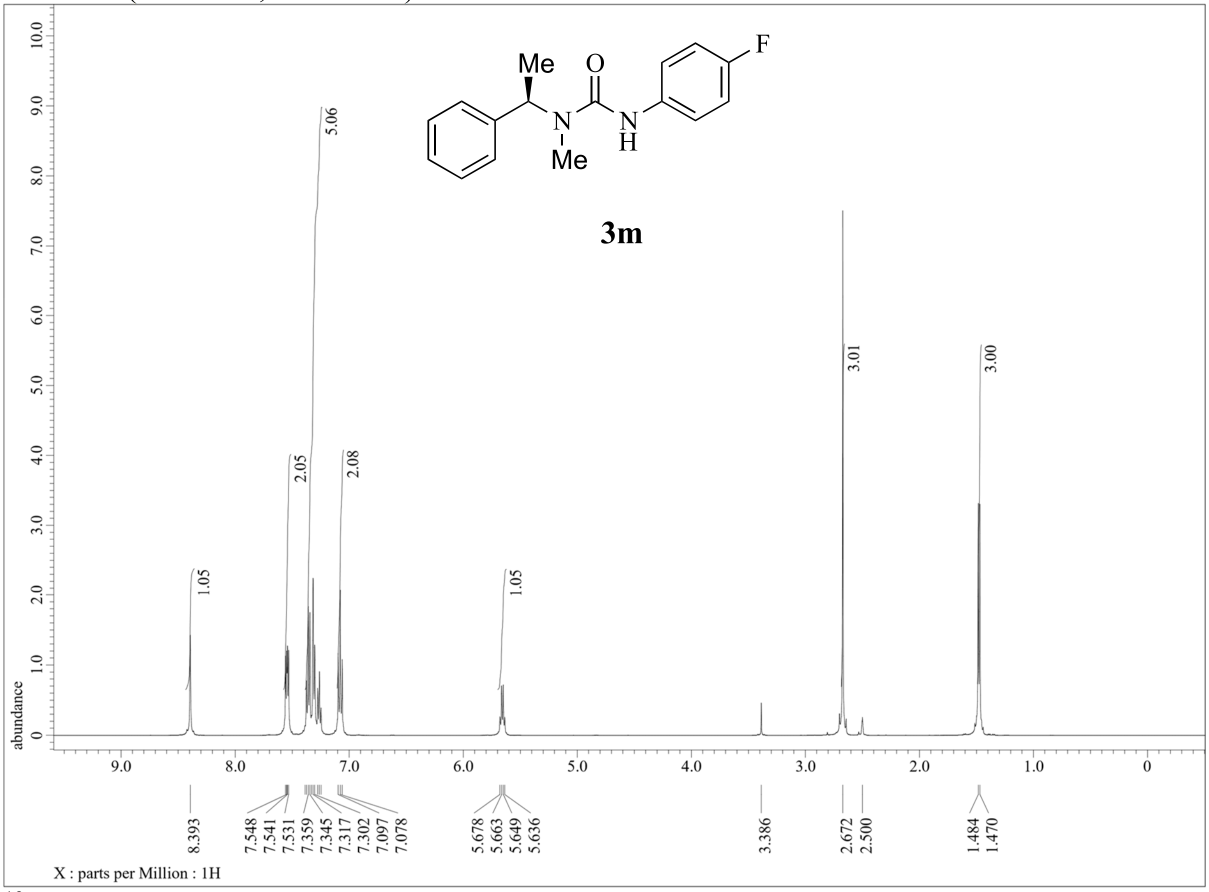

^{13}C NMR (125 MHz, DMSO-d_6)

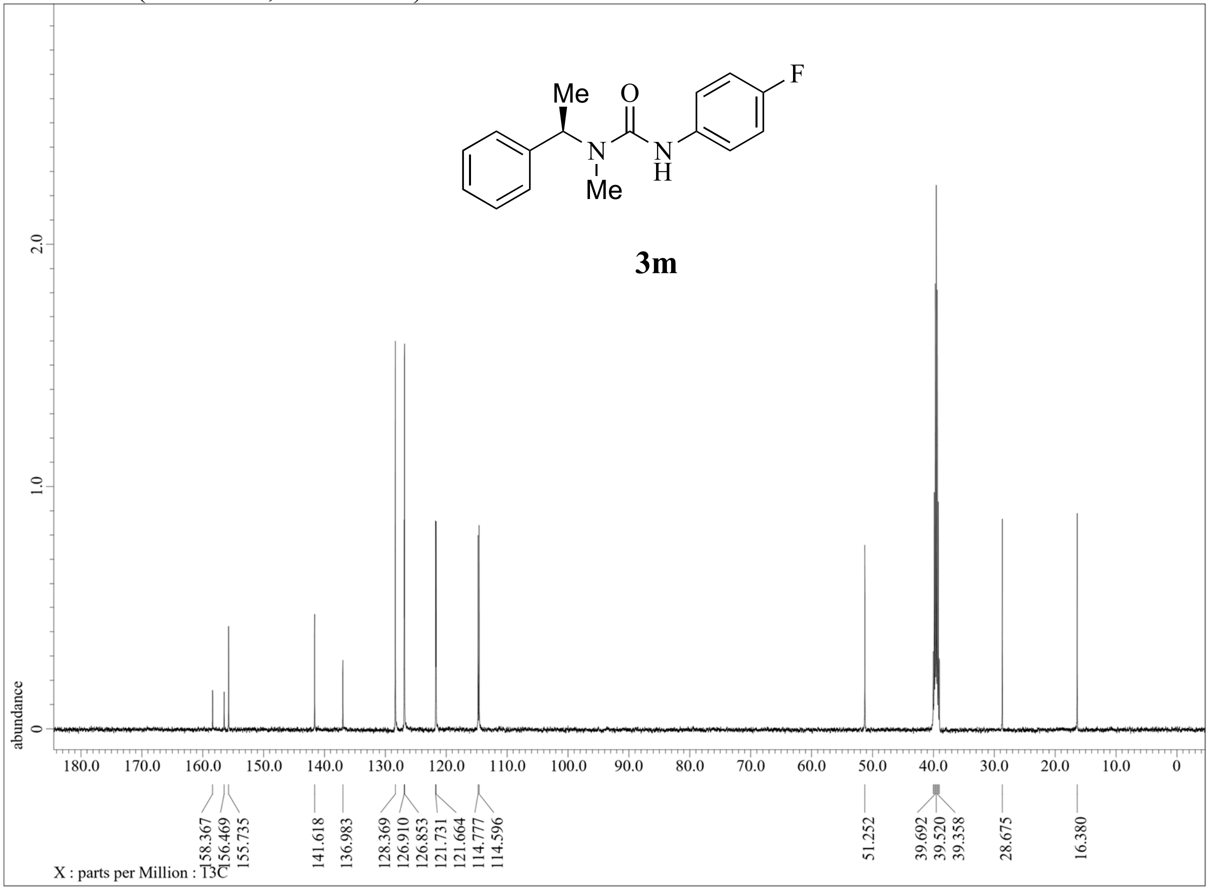

^{1}H NMR (500 MHz, DMSO-d_6)

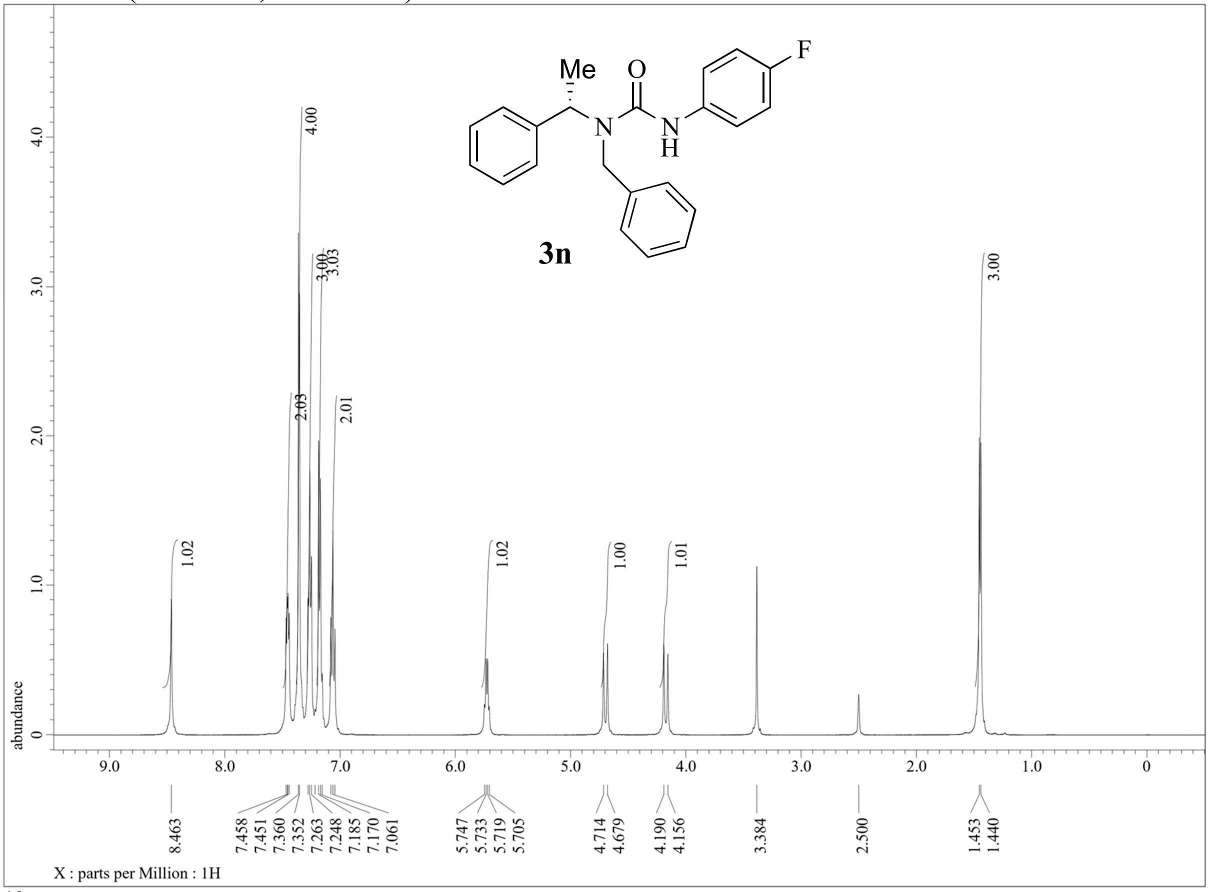

^{13}C NMR (125 MHz, DMSO-d_6)

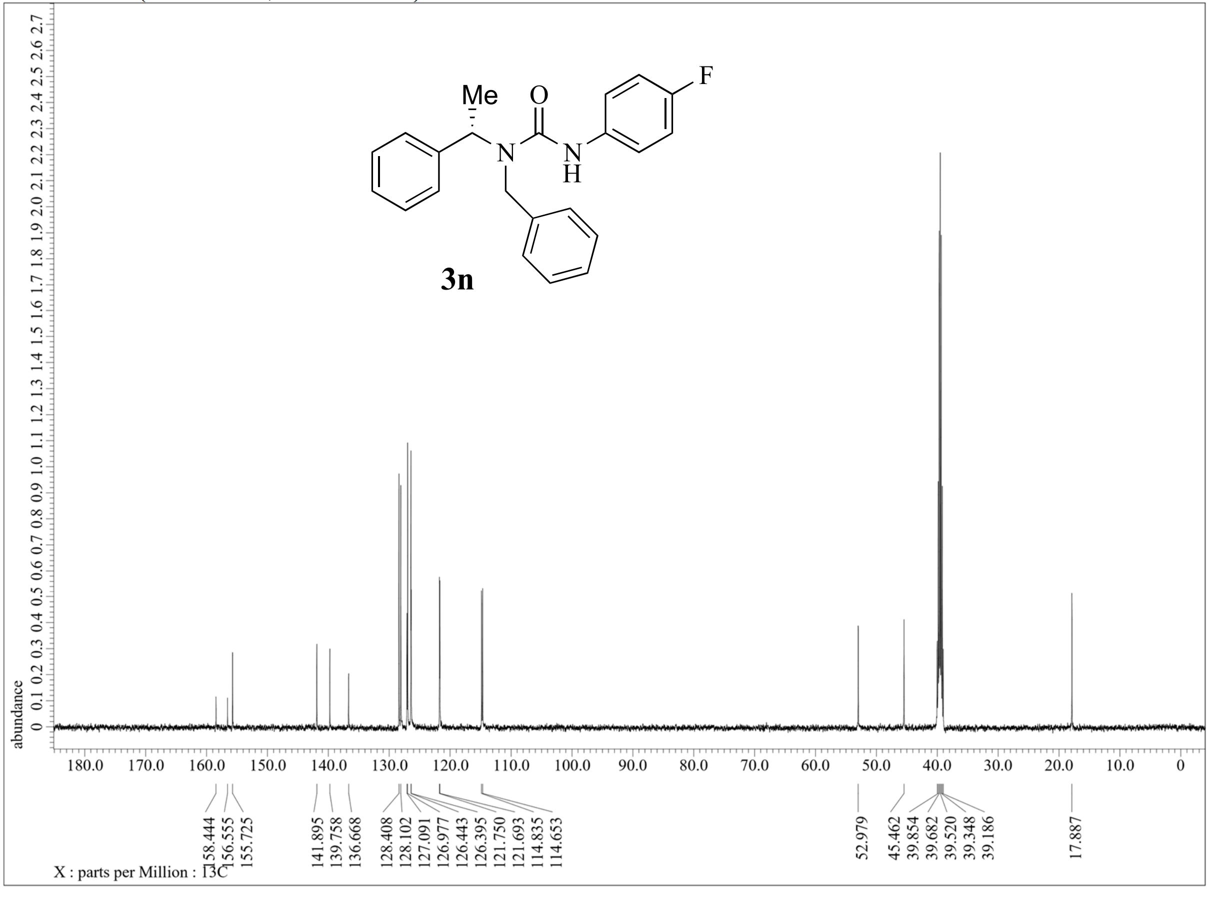

^{1}H NMR (500 MHz, DMSO-d_6)

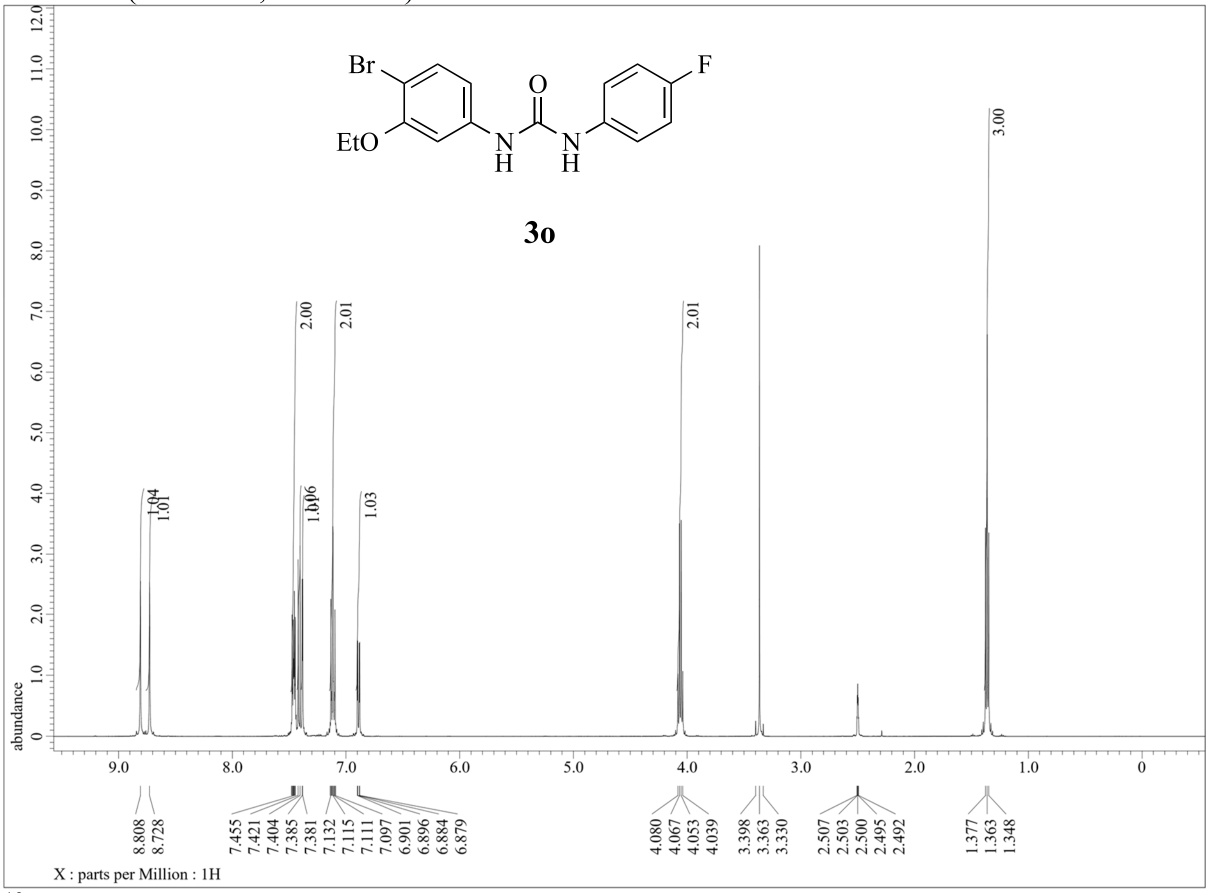

^{13}C NMR (125 MHz, DMSO-d_6)

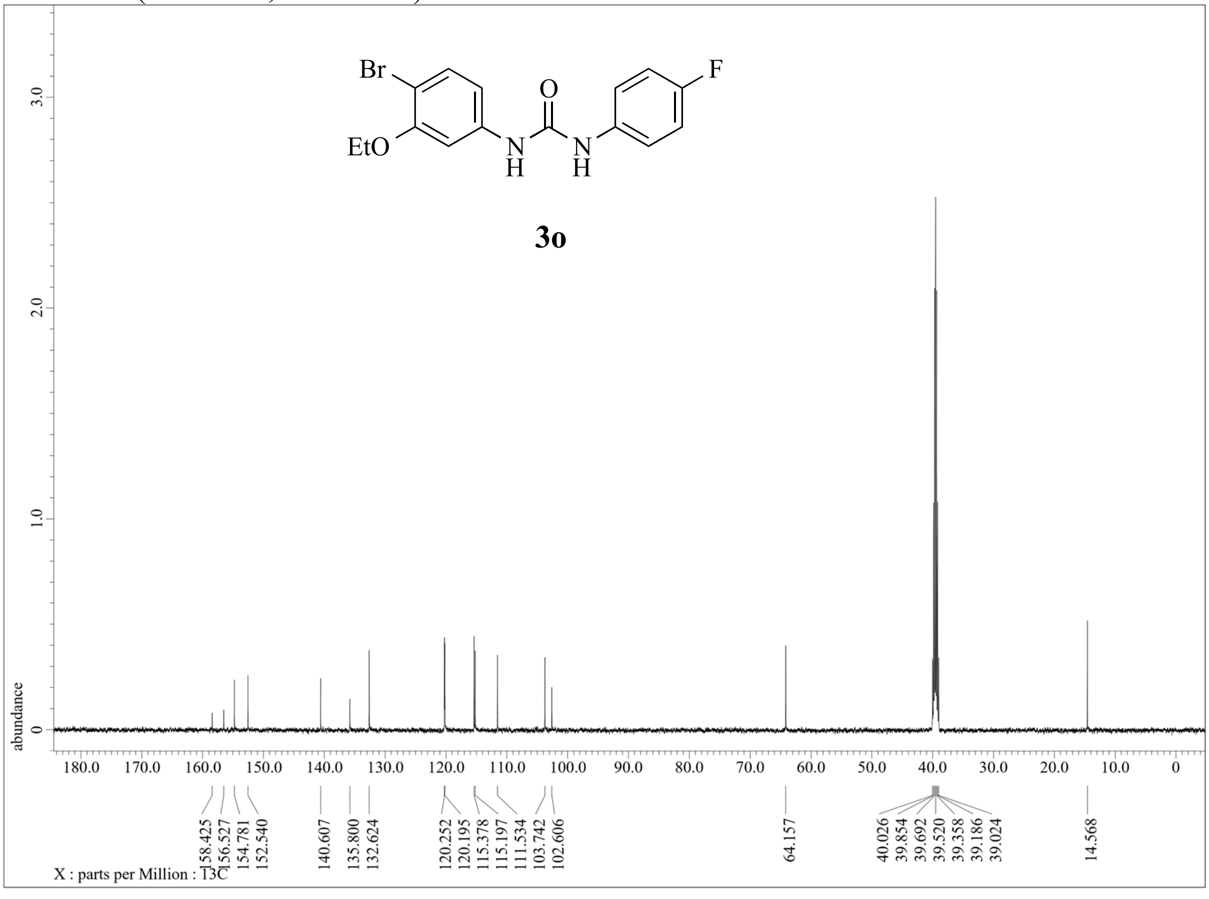

^{1}H NMR (500 MHz, DMSO-d_6)

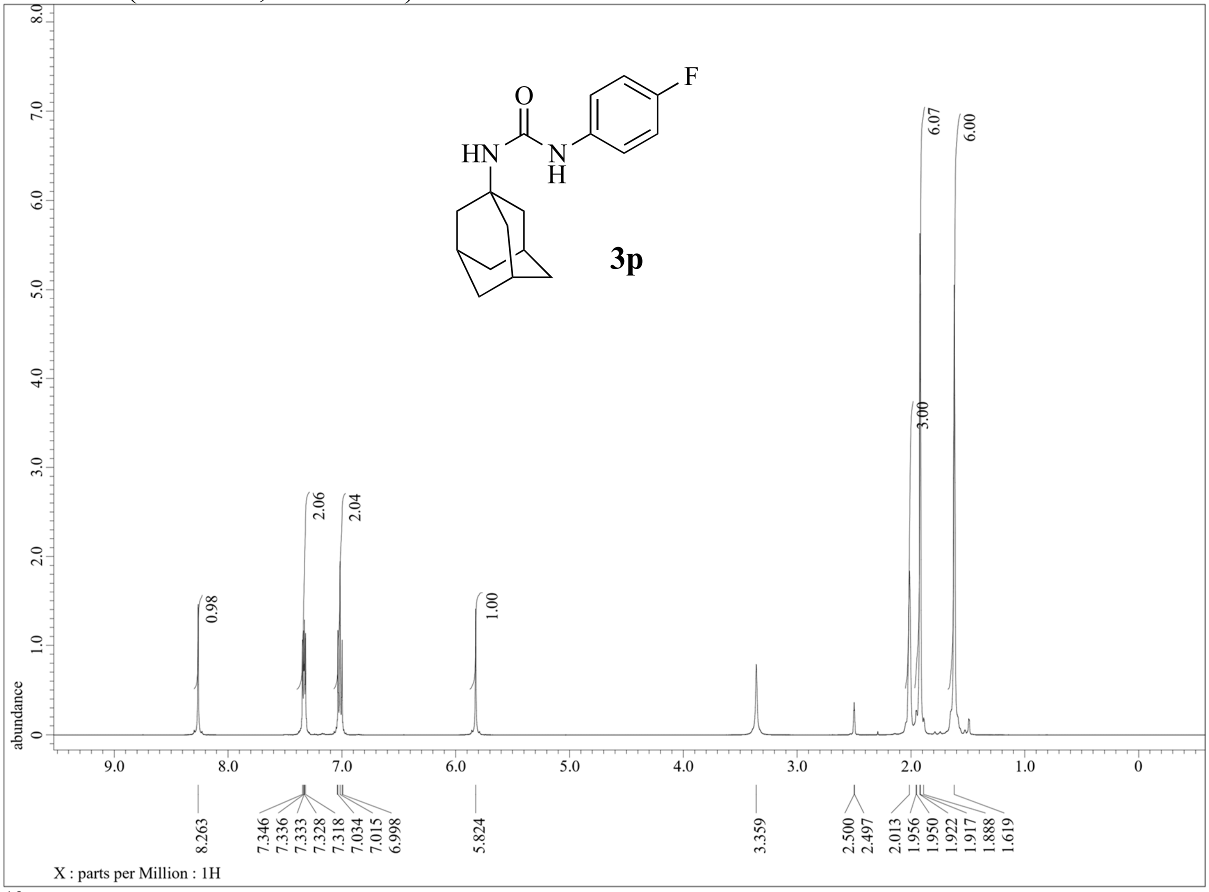

^{13}C NMR (125 MHz, DMSO-d_6)

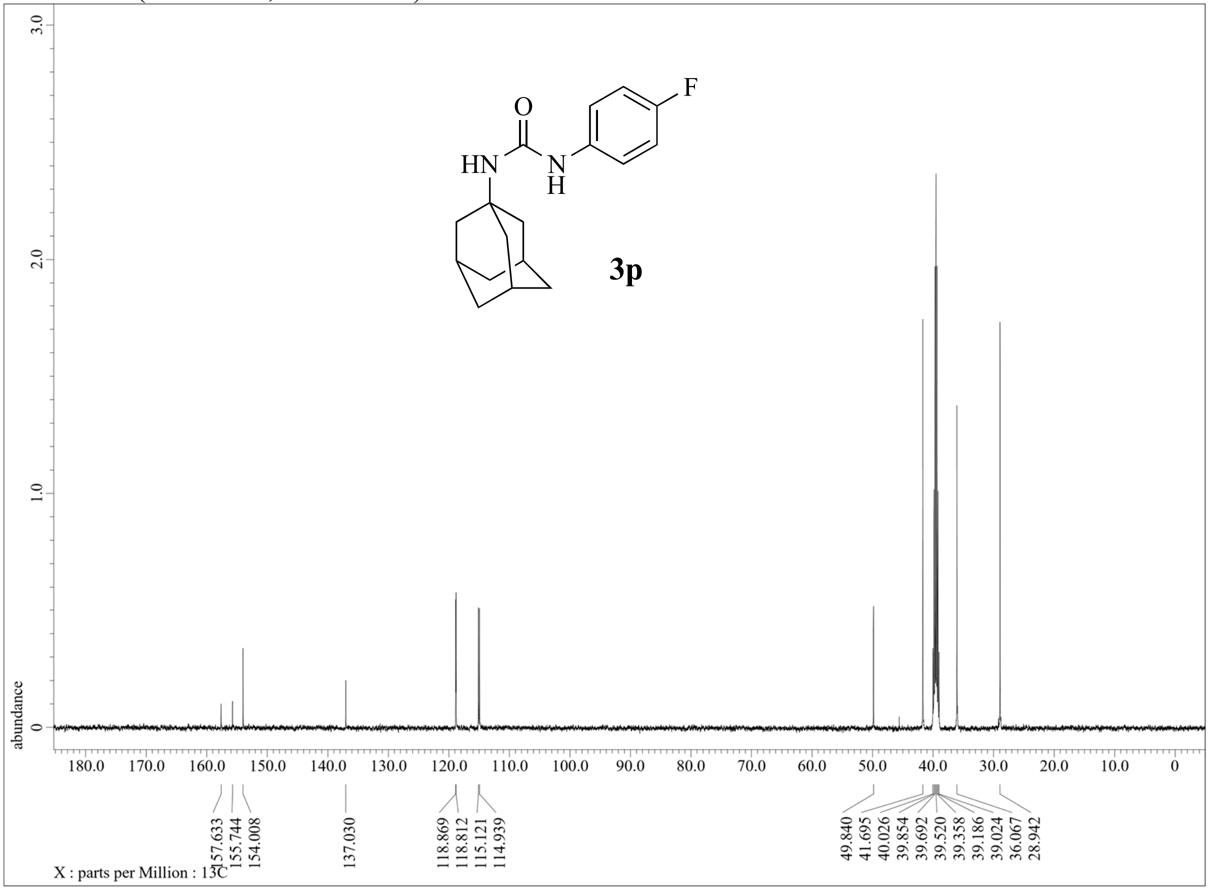

^{1}H NMR (500 MHz, DMSO-d_6)

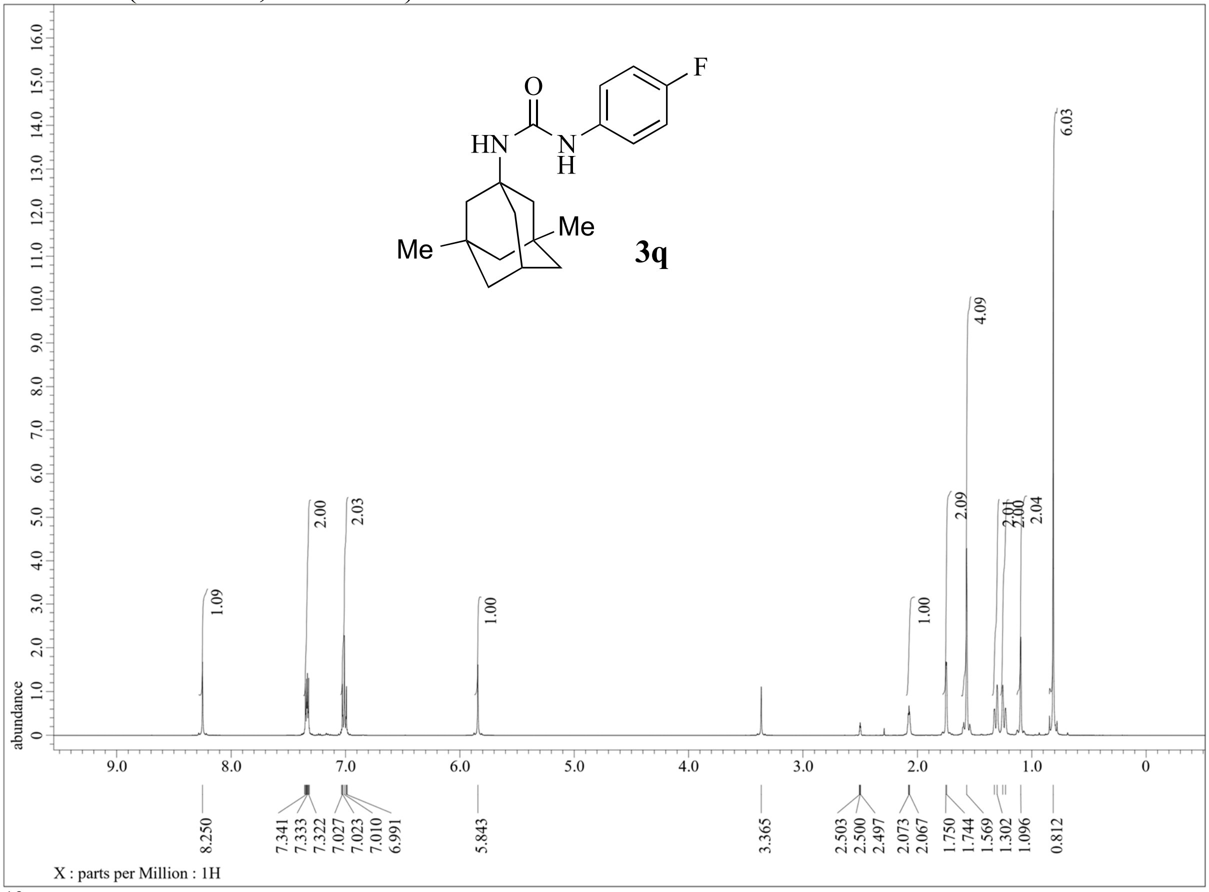

^{13}C NMR (125 MHz, DMSO-d_6)

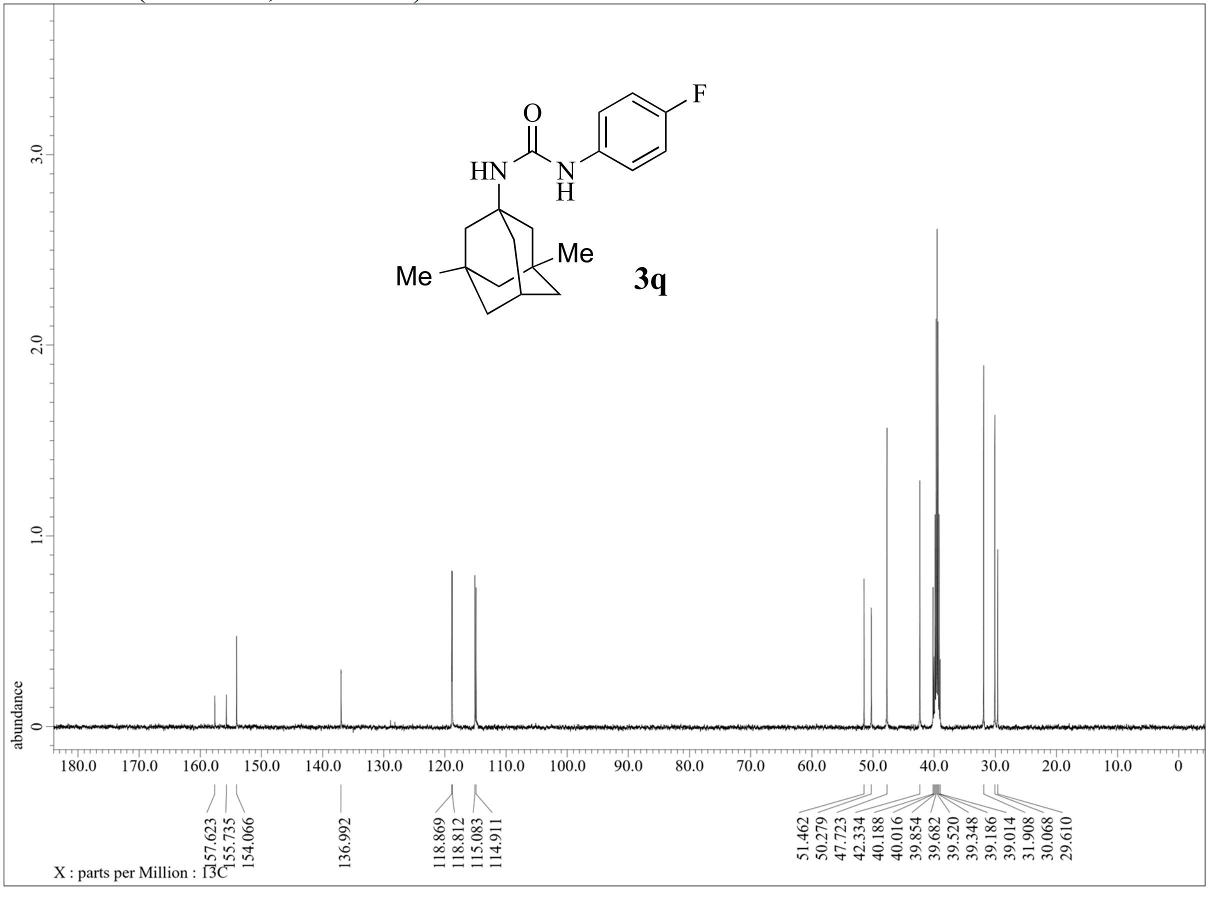

Supplement: Supplementary file 1 [file antibiotics-08-00178-s001.pdf]
